# The S2 Pocket Governs the Genus‐Specific Substrate Selectivity of Coronavirus 3C‐Like Protease

**DOI:** 10.1002/advs.202407766

**Published:** 2024-10-08

**Authors:** Junwei Zhou, Peng Sun, Zhixiang Yang, Taiquan Wang, Jiahui Guo, Runhui Qiu, Zhuang Li, Dengguo Wei, Jinshui Zheng, Guiqing Peng, Liurong Fang, Shaobo Xiao

**Affiliations:** ^1^ State Key Laboratory of Agricultural Microbiology College of Veterinary Medicine Huazhong Agricultural University Wuhan 430070 China; ^2^ The Key Laboratory of Preventive Veterinary Medicine in Hubei Province Cooperative Innovation Center for Sustainable Pig Production Wuhan 430070 China

**Keywords:** 3C‐like protease, coronavirus, drug design, evolutionary trajectory, S2 pocket

## Abstract

Coronavirus 3C‐like protease (CoV 3CL^pro^) is essential for viral replication, providing an attractive target for monitoring the evolution of CoV and developing anti‐CoV drugs. Here, the substrate‐binding modes of 3CL^pro^s from four CoV genera are analyzed and found that the S2 pocket in 3CL^pro^ is highly conserved within each genus but differs between genera. Functionally, the S2 pocket, in conjunction with S4 and S1′ pockets, governs the genus‐specific substrate selectivity of 3CL^pro^. Resurrected ancestral 3CL^pro^s from four CoV genera validate the genus‐specific divergence of S2 pocket. Drawing upon the genus‐specific S2 pocket as evolutionary marker, eight newly identified 3CL^pro^s uncover the ancestral state of modern 3CL^pro^ and elucidate the possible evolutionary process for CoV. It is also demonstrated that the S2 pocket is highly correlated with the genus‐specific inhibitory potency of PF‐07321332 (an FDA‐approved drug against COVID‐19) on different CoV 3CL^pro^s. This study on 3CL^pro^ provides novel insights to inform evolutionary mechanisms for CoV and develop genera‐specific or broad‐spectrum drugs against CoVs.

## Graphical abstract

This study reveals the critical role of the S2 pocket in coronavirus 3C‐like protease (CoV 3CL^pro^) for substrate selectivity and its variability across CoV genera. Analysis of ancestral and modern 3CL^pro^s sheds light on evolutionary processes and their effect on the efficacy of PF‐07321332. These findings offer new insights into CoV evolution and drug development.

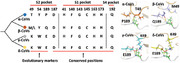

## 1 Introduction

Coronaviruses (CoVs) are enveloped, positive‐sense, single‐stranded RNA viruses that are widely distributed in humans, other mammals, and birds, in which they cause respiratory, intestinal, hepatic, and neurological diseases.^[^ ^1^, ^2^ ^]^ CoVs are members of the order *Nidovirales*, family *Coronaviridae*, and subfamily *Orthocoronavirinae*. Based on genome analysis, the *Orthocoronavirinae* subfamily is further divided into four genera: *Alphacoronavirus* (α‐CoV), *Betacoronavirus* (β‐CoV), *Gammacoronavirus* (γ‐CoV), and *Deltacoronavirus* (δ‐CoV).^[^ ^3^, ^4^ ^]^ To date, seven human coronaviruses (HCoVs) have been identified, namely HCoV‐229E, HCoV‐OC43, HCoV‐NL63, HCoV‐HKU1, severe acute respiratory syndrome coronavirus (SARS‐CoV), Middle East respiratory syndrome coronavirus (MERS‐CoV), and SARS‐CoV‐2.^[^ ^5^, ^6^, ^7^ ^]^ In addition to HCoVs, porcine enteropathogenic CoVs, including transmissible gastroenteritis virus (TGEV), porcine epidemic diarrhea virus (PEDV), porcine deltacoronavirus (PDCoV), and the emerging swine acute diarrhea syndrome coronavirus (SADS‐CoV), are major pathogenic causes of piglet diarrhea and have led to tremendous economic losses to the pork industry.^[^ ^8^, ^9^ ^]^ Recently, a human infection of PDCoV was reported, and the virus was isolated from plasma samples of children with acute febrile illness.^[^ ^10^ ^]^ The emergence and re‐emergence of CoVs‐related epidemics and the increasing evidence for interspecies transmission of CoVs have become the most serious threat to global public health.

The conserved nonstructural protein of CoVs, nsp5 (chymotrypsin‐like main protease, 3CL^pro^; also known as main protease, M^pro^) is mainly responsible for hydrolyzing orf1a and orf1ab into 15–16 mature nonstructural proteins for viral replication and transcription. Moreover, 3CL^pro^ can blunt the immediate antiviral immune response via hydrolyzing the host factors involved in the innate immune response, including the host signal transducer and activator of transcription 2 (STAT2) and the NF‐κB essential modulator (NEMO).^[^ ^11^, ^12^ ^]^ These critical functions make 3CL^pro^ an attractive target for the design of anti‐CoV therapies. CoV 3CL^pro^ inhibitors mimic the interaction between 3CL^pro^ and P4‐P1′ sites (P and P′ denote the residues before and after the scissile bond, respectively) in peptide substrate to gain inhibitory potency and specificity.^[^ ^13^, ^14^ ^]^ Slight alterations in the substrate‐binding mode of CoV 3CL^pro^ might affect inhibitor binding without affecting peptide substrate interactions. However, the molecular mechanisms underlying the diverse substrate specificity of CoV 3CL^pro^ have not been elucidated yet, hampering the design and optimization of genera‐specific and broad‐spectrum inhibitors.

Evolution can generate novel complex molecular structures and functions through elementary genetic mechanisms that incorporate existing biophysical features into higher‐level architectures. Such studies on the nsp5 and spike genes of SARS‐CoV‐2 have yielded valuable insights into the evolutionary selection pressure of SARS‐CoV‐2.^[^ ^15^, ^16^ ^]^ However, constrained by the phylogenetic tree inferred from the 1D nucleotide or amino acid sequence, the evolutionary relationships between different genera within the *Orthocoronavirinae* subfamily have not been extensively investigated. Therefore, a comprehensive study on the structure and function of various CoV 3CL^pro^s represents a promising perspective to elucidate the evolutionary processes of CoVs in the four different genera, and to facilitate the design of antiviral inhibitors.

In this study, six representative 3CL^pro^s from four CoV genera were selected to characterize the substrate‐binding modes. The CoV 3CL^pro^ was found to possess genus‐specific S2 pocket and substrate selectivity. Ancestral sequence reconstruction of ancient 3CL^pro^s from four CoV genera supported the genus‐specific divergence of the S2 pocket. Eight newly described 3CL^pro^s exhibited the diverse S2 pocket and might represent the ancestral state of modern 3CL^pro^. Furthermore, S2 pocket primarily contributes to the genus‐specific inhibitory potency of PF‐07321332 against different CoV 3CL^pro^s. Our in‐depth study of the molecular mechanisms underlying 3CL^pro^ substrate specificity can be used to inform the evolutionary process of CoVs and to develop strategies for the optimization of genus‐specific and broad‐spectrum inhibitors.

## 2 Results

### 2.1 Genus‐Specific Diversity of the S2 Pocket in the CoV 3CL^pro^

**Figure 1:**
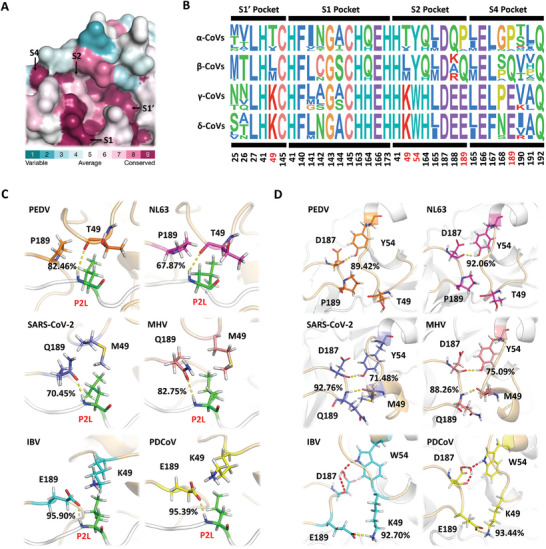
CoV 3CL^pro^ exhibits genus‐specific S2 pocket. A) ConSurf analysis of CoV 3CL^pro^s. The analysis involves the surface mapping of aligned amino acid sequences to the 3D structure of SARS‐CoV‐2 (Protein Data Bank ID 7N89). Highly conserved residues are maroon, residues that evolve at average rates are shown in white, and highly variable residues are blue in color. B) Sequence alignment of amino acids that comprise the S1, S2, S4, and S1′ pockets. C) Diagram of the three distinct substrate‐binding modes in the S2 pocket. Hydrogen bond interactions are shown as yellow dashed lines. Percentage represents the hydrogen bond occupancy between P2 site and residue 49 or 189. D) Detailed interactions of the intra‐molecular networks in S2 pocket of the six 3CL^pro^s. Yellow dashed lines indicate direct electrostatic interaction between the two loops. Red dashed lines indicate the water bridge between W54 and D187 in IBV and PDCoV 3CL^pro^s. Percentage represents the hydrogen bond occupancy of the intra‐molecular interactions in the S2 pocket.

For efficient and specific substrate recognition, the active sites of 3CL^pro^ are composed of four pockets: S1, S2, S4, and S1′. To explore the distinction among various CoV 3CL^pro^s, 44 representative CoVs species from the *Orthocoronavirinae* subfamily in The International Committee on Taxonomy of Viruses (ICTV) were selected. The sequence alignment of all CoV 3CL^pro^s revealed that the primary difference in the active sites was localized in the S2 pocket (**Figure** **1**A). Specifically, compared to β‐CoVs, α‐CoVs lack one amino acid in the loop 41–54 of S2 pocket, while γ‐ and δ‐CoVs lack three amino acids (Figure S1A, Supporting Information). Additionally, the amino acids at positions 49, 54, and 189 in the S2 pocket are T, Y, and P in α‐CoVs, M/L, Y, and Q in β‐CoVs, and K, W, and E in γ‐ and δ‐CoVs (using SARS‐CoV‐2 3CL^pro^ numbering) (Figure 1B). These features are highly conserved within each genus but vary between genera, suggesting the genus‐specific S2 pocket in CoV 3CL^pro^s.

To further investigate the potential diversity of substrate‐binding modes in CoVs, we chose six CoVs from four genera (α‐CoVs: PEDV and NL63, β‐CoVs: SARS‐CoV‐2 and MHV, γ‐CoV: IBV, and δ‐CoV: PDCoV) and performed molecular dynamic (MD) simulations of each 3CL^pro^ in complex with the nsp4/nsp5 autocleavage sequence (TSAVLQ↓SGFRKM) of SARS‐CoV‐2. Ten well‐defined inter‐molecular hydrogen bonds were formed between 3CL^pro^ and the peptide substrate, of which nine hydrogen bonds that formed at P1, P3, P4, and P2′ sites were relatively consistent and conserved among the six 3CL^pro^s (Figure S1B, Supporting Information). In contrast, the hydrogen bonding network in S2 pocket was diverse. The T49 in PEDV and NL63 3CL^pro^s constituted main‐chain interactions with P2‐L. However, in SARS‐CoV‐2 and MHV 3CL^pro^s, the side chain amide group of Q189 formed a hydrogen bond with P2‐L. Intriguingly, the binding modes in IBV and PDCoV 3CL^pro^ S2 pockets were identical, with the side chain carboxylate oxygen atom of E189 providing a stable hydrogen bond receptor for P2‐L (using SARS‐CoV‐2 3CL^pro^ numbering) (Figure 1C). These three binding modes in S2 pocket were also present in the complex system of CoV 3CL^pro^ with NEMO substrate (**aa_226‐237_**) (Figure S1C, Supporting Information). Consistently, the intra‐molecular interactions in S2 pocket also exhibited three distinct modes. In the PEDV and NL63 3CL^pro^s, the two loops (aa 41–54 and 187–190) in S2 pocket were connected and stabilized by a hydrogen bond between Y54 and D187. In SARS‐CoV‐2 and MHV 3CL^pro^s, apart from the analogous hydrogen bond generated between Y54 and D187, M49 established a stable backbone hydrogen bond with Q189. In IBV and PDCoV 3CL^pro^s, a water bridge between W54 and D187 substituted the stable hydrogen bonding network between Y54 and D187 and an exclusive and robust salt bridge was identified between K49 and E189 (Figure 1D). These three distinct inter‐ and intra‐molecular interactions engaged by genus‐specific residues 49, 54, and 189 demonstrated the different substrate‐binding mode in CoV 3CL^pro^ S2 pocket of four different genera.

### 2.2 Genus‐Specific Substrate‐Binding Mode in CoV 3CL^pro^ S2 Pocket Determines the P2 Selectivity

**Figure 2:**
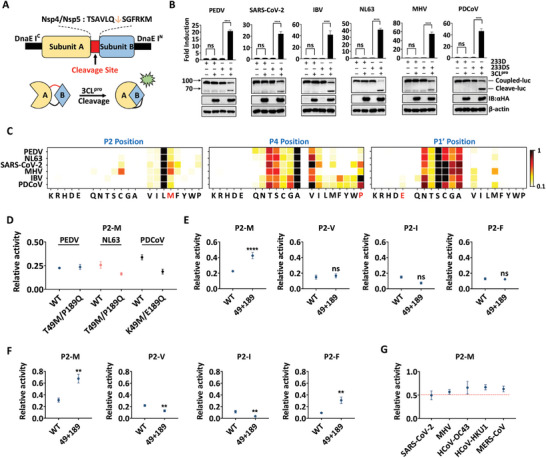
The loops within the S2 pocket determine the substrate preference of CoV 3CL^pro^ at P2 site. A) Diagram showing the generation of luciferase‐based biosensor. The subunit A and B represent the peptide sequence of the recombinant firefly luciferase. The black rectangle represents the Nostoc punctiforme (Npu) DnaE intein (DnaE I) peptide sequence used to cyclize the protein. The red rectangle represents the nsp4/nsp5 autocleavage sequence (TSAVLQ↓SGFRKM) of SARS‐CoV‐2 that was used to assess CoV 3CL^pro^ activity (233DS). The sequence ENLYFQ↓YS, cleaved by the tobacco etch virus (TEV) 3C^pro^, was used to construct the reporter control (233D). B) Reliability of the cyclized luciferase‐based biosensor at detecting the six 3CL^pro^s activity in mammalian cells. The fold induction and the cleavage in each 3CL^pro^ system validated their potential utility in assessing the activity of 3CL^pro^ in HEK‐293T cells. αHA, antihemagglutinin; IB, immunoblotting; luc, luciferase. C) Position scanning peptide libraries were used to determine the substrate specificity of the six CoV 3CL^pro^s at P2, P4, and P1′ sites. The relative cleavage activities of 3CL^pro^ against these substrate sequences were determined by a high‐throughput luciferase‐based biosensor as previously described. Red indicates a preference for a given amino acid, while white indicates counter selection. D) Relative protease activity of various 3CL^pro^ X49M/X189Q mutants against P2‐M substrate. E) Relative protease activity of PEDV 3CL^pro^ double‐loop substitution mutant against P2‐M, V, I, and F substrates. F) Relative protease activity of PDCoV 3CL^pro^ double‐loop substitution mutant against P2‐M, V, I, and F substrates. G) Relative protease activity of β‐CoV 3CL^pro^s against P2‐M substrate.

Substrate selectivity is highly regulated by specific well‐defined interactions between protease and substrate, making it a functional representation of substrate‐binding mode. Therefore, eleven putative cleavage sites of 3CL^pro^ were predicted based on the alignment of pp1ab polyproteins from all 44 CoVs species. Substrate selectivity at P1, P2, P4, and P1′ sites was relatively conserved, which corresponded to four pockets in the active sites of 3CL^pro^ (Figure S1D, Supporting Information). To further characterize the substrate selectivity of CoV 3CL^pro^, a luciferase‐based biosensor was employed to evaluate the cleavage activity of 3CL^pro^ in mammalian cells against different substrates (**Figure** **2**A). All six 3CL^pro^s recognized and cleaved the nsp4/nsp5 autocleavage sequence (TSAVLQ↓SGFRKM) of SARS‐CoV‐2 3CL^pro^ fused in the luciferase reporter plasmid. The fold induction and the cleavage in each 3CL^pro^ system validated their potential utility in assessing the activity of 3CL^pro^ in HEK‐293T cells (Figure 2B). Given the unambiguous preference for Gln at P1 site, we performed saturation mutagenesis at P2, P4, and P1′ sites in the nsp4/nsp5 autocleavage sequence and the cleavage activities of six proteases against different substrates were determined. Consistent with the alignment of autocleavage sequences, the P2 site was the most restrictive, followed by P1′ and P4 sites (Figure 2C). Specifically, the P2 site favored hydrophobic residues (L, M, V, F, and I). Compared to the other four 3CL^pro^s, SARS‐CoV‐2 and MHV 3CL^pro^s were more tolerant to amino acids with larger side chains at the P2 site, particularly Met. All six 3CL^pro^s maintained favorable relative activity for substrates with small side chains at the P4 site. However, tolerance for amino acids with large side chains (such as P, Y, F, and W) at P4 site was found to be more pronounced in SARS‐CoV‐2, MHV, IBV, and PDCoV 3CL^pro^s than PEDV and NL63 3CL^pro^s. The P1′ site possessed a preference for amino acids with small side chains, such as S, A, G, and C. Our previous study showed that PDCoV 3CL^pro^ cleaved STAT2 and the cleavage motif contained a nonclassical Glu at P1′ site.^[^^]^ Interestingly, similar to PDCoV, IBV could also cleave substrate with P1′‐E (Figure 2C). Overall, the substrate selectivity of the six CoV 3CL^pro^s at P2, P4, and P1′ sites was broadly consistent, but some subtle diversity was also present, showing high genus specificity.

To investigate the effect of the genus‐specific S2 pocket on substrate selectivity, MD simulations of each protease with P2‐M substitution substrate were performed. After introducing Met at P2 site, the T49, K49, and E189 in S2 pocket of PEDV, NL63, IBV, and PDCoV 3CL^pro^ gradually shifted away from P2‐M, which completely disrupted the hydrogen bonding networks within S2 pocket compared to the wild type (WT) systems (Figure S2A,D, Supporting Information). Therefore, we determined the relative activity of PEDV, NL63, and PDCoV 3CL^pro^ X49M/X189Q mutants against substrates with M, V, F, or I at the P2 site. Although the amino acids at positions 49 and 189 have been replaced with Met and Gln, respectively, mimicking SARS‐CoV‐2 and MHV 3CL^pro^s, their tolerance for amino acids with bulky side chains at the P2 site has not been significantly enhanced (Figures 2D and S3A,D, Supporting Information). Furthermore, the two loops involving residues 41–54 and 187–190 in S2 pocket of PEDV, NL63, IBV, and PDCoV 3CL^pro^ were substituted with that of SARS‐CoV‐2 3CL^pro^. Compared to their respective single‐loop substitution mutants, PEDV and PDCoV 3CL^pro^ double‐loop substitution mutants recovered moderate protease activity (Figure S3E, Supporting Information), suggesting the potential formation of a stable S2 pocket. Both mutants displayed enhanced tolerance for amino acids with large side chains at the P2 site, particularly Met (Figure 2E,F). The MD simulations indicated that the classical intramolecular interactions within the S2 pocket of SARS‐CoV‐2 and MHV 3CL^pro^s were reconstructed in PEDV and PDCoV 3CL^pro^ double‐loop substitution mutants (Figure S3F,G, Supporting Information). Notably, the artificially replaced networks, especially in PDCoV 3CL^pro^, were not as stable as those in SARS‐CoV‐2 and MHV 3CL^pro^s, which might account for their somewhat reduced protease activity (Figure S3H, Supporting Information). In addition, MERS‐CoV, HCoV‐HKU1, and HCoV‐OC43 3CL^pro^s with similar β‐genus characteristics in S2 pocket also exhibited favorable relative activity against P2‐M substrate (Figure 2G). These results suggested that the 41–54 and 187–190 loops in CoV 3CL^pro^, composed of different amounts of residues with distinct properties, give rise to the genus‐specific S2 pocket and substrate selectivity.

### 2.3 Synergistic Effect of S2 and S4 Pockets in Substrate Specificities

**Figure 3:**
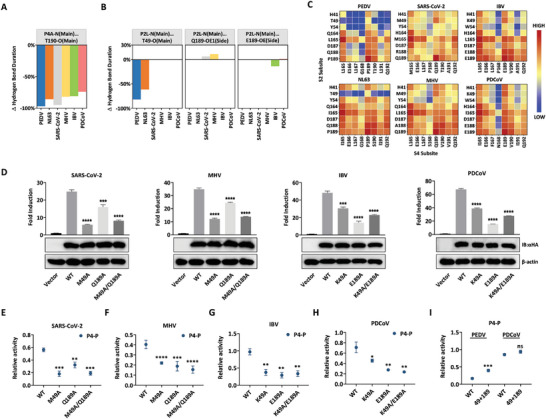
Correlation between S2 and S4 pockets affected the substrate selectivity at P4 site. A) Variation of hydrogen bond occupancy in the S4 pocket for P4‐P substitution system relative to the WT system. B) Variation of hydrogen bond occupancy in the S2 pocket for P4‐P substitution system relative to the WT system. C) Cross‐correlations between atomic fluctuations of residues in the six CoV 3CL^pro^ S2 and S4 pockets. Warm colors in the matrices indicate increased correlations. D) Protease activity of the 3CL^pro^ X49A, X189A, and X49A/X189A mutants. αHA, antihemagglutinin; IB, immunoblotting. E–H) Relative protease activity of 3CL^pro^ mutants against P4‐P substrate. I) Relative protease activity of PEDV and PDCoV 3CL^pro^ double‐loop substitution mutants against P4‐P substrate.

Compared to PEDV and NL63 3CL^pro^s from α genus, the other four 3CL^pro^s from β, γ, and δ genera displayed a considerably broader substrate selectivity at the P4 site, particularly for Pro. To elucidate the molecular mechanism under the selectivity of CoV 3CL^pro^s at P4 site, MD simulations of each protease with P4‐P substitution substrate were performed. The introduction of Pro at P4 site not only disrupted the main chain hydrogen bond in S4 pocket, but also destabilized the S2 pocket in the PEDV‐ and NL63‐P4‐P systems (**Figures** **3**A,B and S4, Supporting Information), implying that an irrational substitution at P4 site would affect the binding mode in S2 pocket. To further investigate the coupling of atomic fluctuations between S2 and S4 pockets, we calculated the cross‐correlation coefficients between the atomic fluctuations of the backbone in two pockets. In all six P4‐A systems, S2 pocket displayed strong positive (C*_ij_* > 0.8) correlations with S4 pocket, especially with residues 164, 165, and 187–189 (Figure 3C). Notably, the dynamics of S4 pocket were strongly coupled with M49 or K49 in SARS‐CoV‐2, MHV, IBV, and PDCoV S2 pocket but were weakly correlated with T49 in the PEDV and NL63 S2 pocket, which was consistent with the diverse intramolecular interactions between residues 49 and 189 (Figure 1D). Therefore, we speculated that the strong positive correlation induced by the well‐defined interactions between residues 49 and 189 better stabilized the interactions within S2 pocket after the disruption of the S4 pocket network.

To verify this conjecture derived from simulations, we introduced alanine mutations at positions 49 and 189 in SARS‐CoV‐2, MHV, IBV, and PDCoV 3CL^pro^s, attempting to disrupt the binding patterns between residues 49 and 189 in S2 pocket. Mutation at residues 49 or/and 189 decreased the protease activity of these 3CL^pro^s to different extents (Figure 3D). We therefore determined the relative activities of the X49A, X189A, and X49A/X189A mutants against P4‐P substrate. Mutation at residue 49 in S2 pocket reduced the tolerance of SARS‐CoV‐2, MHV, IBV, and PDCoV 3CL^pro^s for Pro at P4 site to a certain extent (Figure 3E,H). Mutation at residue 189, which is shared by S2 and S4 pockets, also reduced the protease activity on P4‐P substrate (Figure 3E,H). Additionally, the S2 pocket substitution significantly strengthened the relative activity of PEDV 3CL^pro^ for P4‐P substrates, whereas showing no effect on PDCoV 3CL^pro^ (Figure 3I). These results confirmed the high cooperativity between S2 and S4 pockets in CoV 3CL^pro^s and demonstrated that the intramolecular interactions between residues 49 and 189 in the S2 pocket affect the selectivity of CoV 3CL^pro^ at P4 site.

### 2.4 CoV 3CL^pro^ S2 Pocket Cooperates with S1′ Pocket for the Substrate Selectivity at P1′ Site

**Figure 4:**
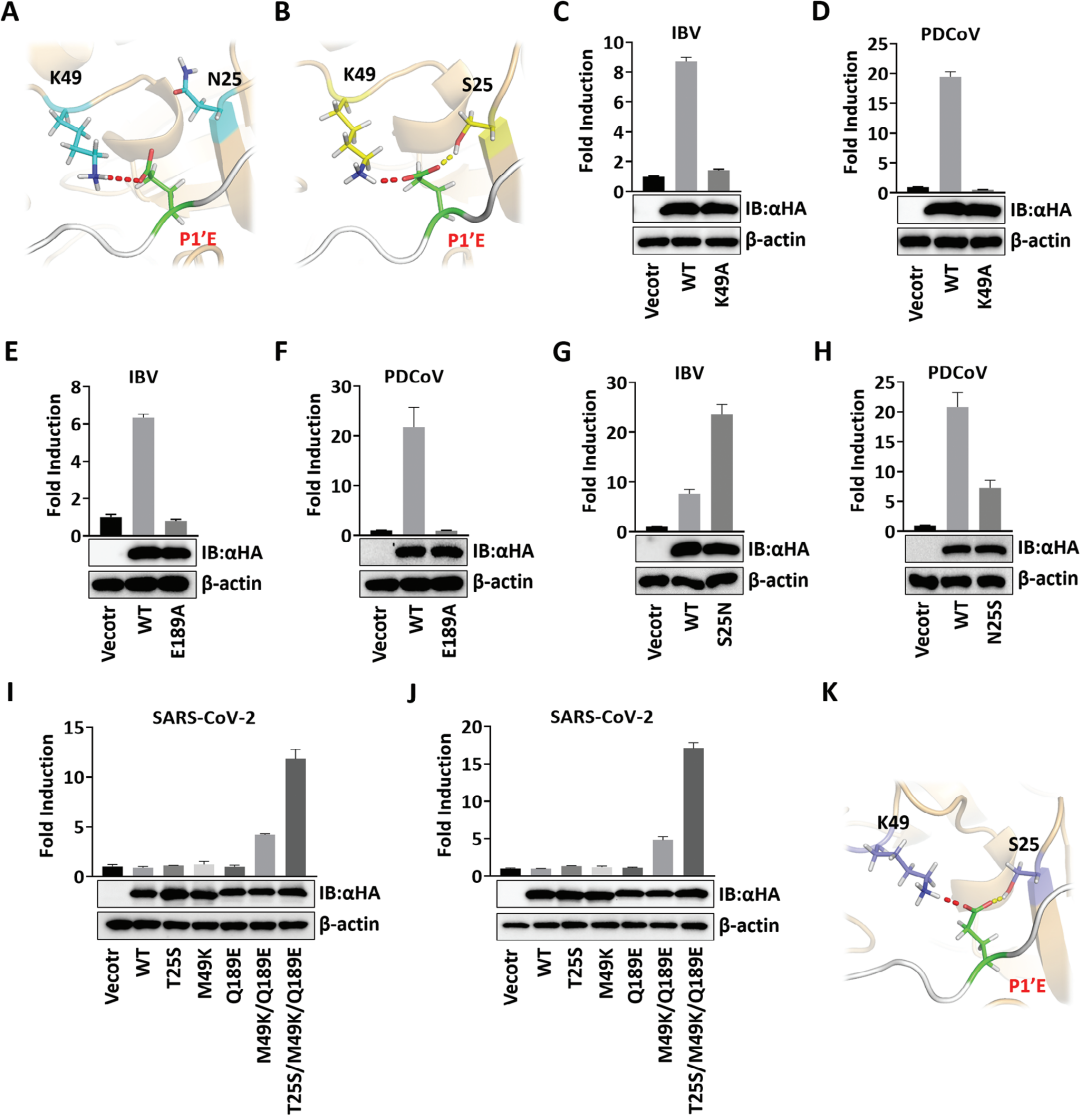
Salt bridge between K49 and E189 is necessary for cleavage to P1′‐E substrate. A–B) Schematic diagram of the specific binding mode in the A) IBV‐ and B) PDCoV ‐P1′‐E systems. Red dashed lines indicate the salt bridge. Yellow dashed lines represent the hydrogen bond. C–H) Protease activity of the WT or 3CL^pro^ mutants for P1′‐E substrate C) for IBV K49A, D) PDCoV K49A, E) IBV E189A, F) PDCoV E189A, G) IBV N25S, and H) PDCoV S25N. aHA, antihemagglutinin; IB, immunoblotting. I) Protease activity of the five SARS‐CoV‐2 3CL^pro^ mutants against P1′‐E substrate. J) Protease activity of the five SARS‐CoV‐2 3CL^pro^ mutants against substrate EKANLQERKKYL. K) Schematic representation of the detailed substrate‐binding mode in the S1′ pocket of SARS‐CoV‐2 triple mutant in complex with P1′‐E substrate. Red dashed line indicates the salt bridge. Yellow dashed line represents the hydrogen bond.

Only IBV and PDCoV 3CL^pro^s recognized and cleaved the substrate with P1′‐E (Figure 2C). In the simulation of each P1′‐E substitution complex, the fraction of main chain hydrogen bonds between residue 26 and P2′‐G was dramatically reduced. More crucially, the oxyanion hole close to P1′ site was also significantly disrupted (Figure S5A, Supporting Information), which could explain the low activity or even complete inactivation of CoV 3CL^pro^ against P1′‐E substrate. However, a novel and stable salt bridge between K49 and P1′‐E was detected in IBV‐ and PDCoV‐P1′‐E complexes (**Figure** **4**A,B). In addition, the oxygen atom of the carboxyl group in P1′‐E formed a hydrogen bond with S25 in the PDCoV‐P1′‐E system (Figure 4B).

To investigate the effect of specific substrate‐binding mode on cleavage activity for P1′‐E substrate, substitution mutagenesis analysis of natural variation within 3CL^pro^s of four genera was performed. The K49A substitution in IBV and PDCoV 3CL^pro^s undermined the ability to cleave P1′‐E substrate (Figure 4C,D). The cleavage activity against P1′‐E substrate was also undetectable in IBV and PDCoV 3CL^pro^ E189A mutants (Figure 4E,F). Interestingly, the efficiency of IBV N25S mutant to cleave P1′‐E substrate was significantly increased compared to the WT, whereas the opposite phenomenon was aroused in PDCoV S25N mutant (Figure 4G,H). Moreover, using SARS‐CoV‐2 3CL^pro^ as a template, a series of mutations were designed to mimic the binding mode of PDCoV 3CL^pro^. The double point M49K/Q189E mutant and the triple T25S/M49K/Q189E mutant gained the ability to cleave P1′‐E substrate among the five mutants (Figures 4I and S5B, Supporting Information). The cleavage activity was significantly enhanced by the introduction of T25S mutation in the double point mutant. Both mutants also recognized the cleavage motif of PDCoV 3CL^pro^ against STAT2 (EKANLQ_685_ERKKYL) (Figure 4J). Meanwhile, the specific binding modes in PDCoV 3CL^pro^ S2 and S1′ pockets were reproduced in the SARS‐CoV‐2 triple mutant (Figures 4K and S5C,E, Supporting Information). These results suggested that the stable salt bridge between K49 and E189 in S2 pocket is necessary for the cleavage against P1′‐E substrate, and residue 25 in the S1′ pocket can affect the hydrolysis rate.

### 2.5 CoV 3CL^pro^ S2 Pocket Represents an Evolutionary Marker

**Figure 5:**
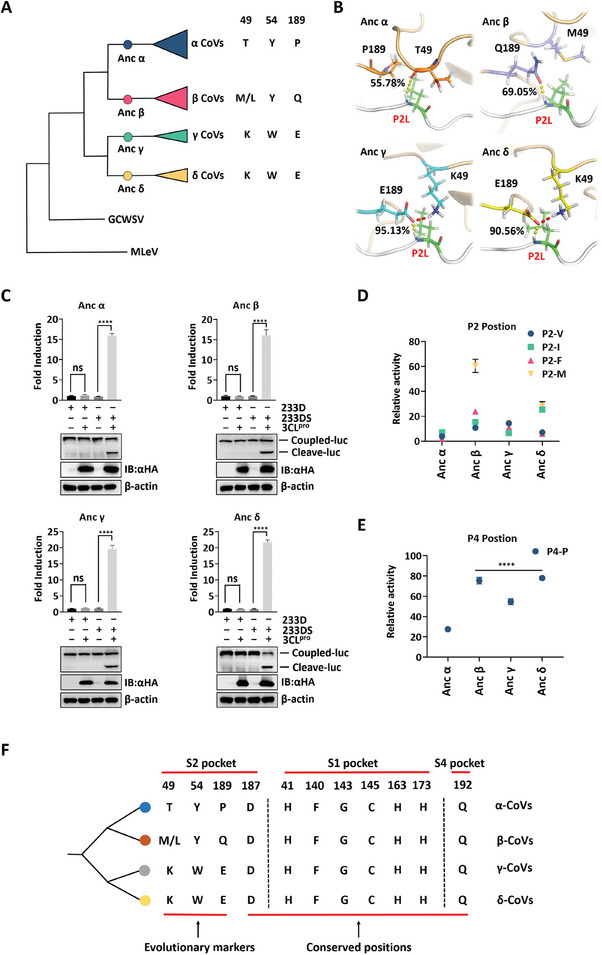
Four ancestral 3CL^pro^s validate the genus‐specific S2 pocket and substrate selectivity of CoV 3CL^pro^. A) Simplified phylogenetic tree of 3CL^pro^ in the *Coronaviridae*. Milecovirus (MLeV, accession numbers: GECV01031551) and Guangdong Chinese water skink coronavirus (GCWSV, accession numbers: AVM87573.1) are new coronaviruses discovered in amphibians and reptiles, respectively. MLeV 3CL^pro^ from the *Letovirinae* subfamily was used as the outgroup to root the tree. The positions of the common ancestor of α‐CoVs, β‐CoVs, γ‐CoVs, or δ‐CoVs, are indicated by circles. B) Schematic representation of the detailed substrate‐binding modes in the four ancestral CoV 3CL^pro^ S2 pockets. Percentage represents the hydrogen bond occupancy between P2 site and residue 49 or 189. C) HEK‐293T cells in 24‐well plates were transfected with the nsp4/nsp5 autocleavage sequence in SARS‐CoV‐2 3CL^pro^ (233DS) or the reporter control (233D). The fold induction and the cleavage in each ancestral 3CL^pro^ system confirmed the cleavage activity of the four reconstructed ancestral CoV 3CL^pro^s against the nsp4/nsp5 autocleavage sequence of SARS‐CoV‐2. D) Relative protease activity of the four ancestral CoV 3CL^pro^s against P2‐M, V, I, F substrates. E) Relative protease activity of the four ancestral CoV 3CL^pro^s against P4‐P substrate. F) Simplified schematic of specific amino acids in the active site of CoV 3CL^pro^. The classification of CoVs based on residues 49, 54, and 189 is consistent with the results derived from ICTV taxonomy database.

To eliminate the sampling bias introduced by the six 3CL^pro^s, we applied ancestral sequence reconstruction to infer the ancient 3CL^pro^ sequences of four CoV genera, namely Anc α, Anc β, Anc γ, and Anc δ (**Figure** **5**A). The ancestral proteins shared the same protein backbone as the modern CoV 3CL^pro^s (Figure S6A, Supporting Information). Three distinct inter‐ and intramolecular interactions were reproduced in the S2 pocket of four ancestral proteins, with Anc α corresponding to PEDV and NL63, Anc β corresponding to SARS‐CoV‐2 and MHV, and Anc γ and δ corresponding to IBV and PDCoV (Figures 5B and S6B,D, Supporting Information). We further synthesized the sequences and tested the cleavage activity. All four ancestral proteins recognized and cleaved the nsp4/nsp5 autocleavage sequence, suggesting that an active site similar to that of modern CoV 3CL^pro^ was restored (Figure 5C). Functionally, Anc β was significantly more tolerant to amino acids with bulky side chains than Anc α, γ, and δ at P2 site, especially to Met (Figure 5D). In addition to Anc α, Anc β, γ, and δ maintained favorable relative activity on substrate with P4‐P (Figure 5E). These results demonstrated that the functional characteristics of S2 pocket were in place by the ancestor of each genus.

Across the *Orthocoronavirinae* subfamily, residues 49, 54, and 189 in S2 pocket possess genus‐specific evolutionary features (Figure 5F) and determine the genus‐specific binding mode and substrate selectivity of CoV 3CL^pro^. Furthermore, these three amino acids in the ancestral 3CL^pro^s also exhibit the characteristics of each genus (Figures 5B and S6D, Supporting Information). Accordingly, they represent one of the best candidates for discrete evolutionary markers to subdivide CoV families into distinct lineages and guide evolutionary routes.

### 2.6 The Newly Discovered 3CL^pro^s in Nonmammalian Aquatic Vertebrates Represent the Ancestral State of CoV 3CL^pro^

**Figure 6:**
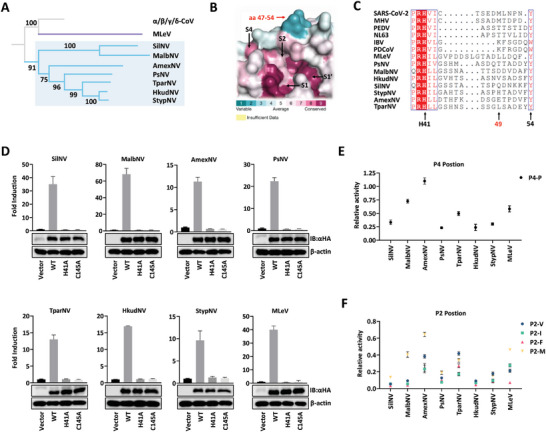
The newly identified 3CL^pro^s exhibit a substrate‐binding mode similar to that of the modern 3CL^pros^. A) Phylogram for the eight newly identified 3CL^pro^ sequences. Four viruses similar to PsNV were in Ambystoma mexicanum (axolotl; AmexNV from read archives SRR6788790), Hippocampus kuda (seahorse; HkudNV from read archives SRR1324965), Syngnathus typhle (broad‐nosed pipefish; StypNV from read archives ERR3994223), and Takifugu pardalis (fugu fish; TparNV from read archives SRR10917299). More distant members identified were in Silurus sp. (catfish; SilNV from read archives SRR12184956), Hypomesus transpacificus (the endangered delta smelt; HtraNV from read archives SRR8389791), and MLeV. B) ConSurf analysis of the eight novel 3CL^pro^s. The analysis involves the surface mapping of aligned amino acid sequences to the 3D structure of SARS‐CoV‐2 (Protein Data Bank ID 7N89). Highly variable residues are blue in color, and highly conserved residues are maroon, residues that evolve at average rates are shown in white. C) The sequence alignment of the 41–54 loop of the eight newly identified 3CL^pro^s and the six selected 3CL^pro^s. D) HEK‐293T cells in 24‐well plates were transfected with 233DS, along with the indicated 3CL^pro^ expression plasmids and pRL‐TK plasmid. E) Relative protease activity of the eight newly identified 3CL^pro^s against P4‐P substrate. F) Relative protease activity of the eight newly identified 3CL^pro^s against P2‐M, V, I, and F substrates.

For the *Coronaviridae* family, the previously known hosts were mammals and birds. Driven largely by the increased availability of high‐throughput sequencing, several novel CoVs have been identified in fish, reptiles, and amphibians.^[^ ^17^, ^18^ ^]^ To explore the evolutionary route of CoV 3CL^pro^, we searched the RNA sequence read archives corresponding to the novel CoVs against a nucleotide sequence of SARS‐CoV‐2 3CL^pro^ and identified six novel 3CL^pro^s. Additionally, we determined the 3CL^pro^s of MLeV and Pacific salmon nidovirus (PsNV) based on the orf1a sequence from the NCBI database. These novel 3CL^pro^s represented a distinct clade sister to the *Orthocoronavirinae* subfamily (**Figure** **6**A). The viruses corresponding to the newly described 3CL^pro^s were mainly found in the gill, whereas members of the *Orthocoronavirinae* subfamily dominated in the lung, which argued for their antiquity. Compared to modern 3CL^pro^s, amino acids constituting the active sites of the newly identified 3CL^pro^s were well conserved, except S2 pocket (Figure 6B). Specifically, the catalytic dyad (H41 and C145) along with F140, H163, and D187 in substrate‐binding pockets exhibited a high degree of conservation (using SARS‐CoV‐2 3CL^pro^ numbering) (Figure S7A, Supporting Information). The 41–54 loop in the novel 3CL^pro^s lacked a discernible pattern and included an unusual amino acid insertion (Figure 6C). We further predicted the autocleavage sequence of 3CL^pro^ in the pp1ab polyproteins from these eight viruses. Similar to modern 3CL^pro^, the eight newly discovered 3CL^pro^s possessed the most restricted substrate specificity at P1 and P2 sites, followed by P4 and P1′ sites (Figure S7B, Supporting Information).

To verify the biological function of these newly identified 3CL^pro^s, the cleavage activity of these proteases was determined. All eight 3CL^pro^s recognized and cleaved the nsp4/nsp5 autocleavage sequence of SARS‐CoV‐2 and employed the same Cys‐His catalytic dyad to hydrolyze the substrate (Figure 6D). We further characterized the selectivity of these eight 3CL^pro^s at P2 and P4 sites. MalbNV, AmexNV, TparNV, and MLeV 3CL^pro^s held a high tolerance toward Pro at P4 site and amino acids with bulky side chains at the P2 site (Figure 6E,F). Consistent with modern 3CL^pro^, the relative activities of these eight 3CL^pro^s against P2‐M and P4‐P substrates are significantly correlated (*R* ^2^ = 0.88), suggesting the coupling of their S2 and S4 pockets as well. It is worth noting that the evolutionary markers residue 49 and 54 in PsNV (T and Y) and MLeV (L and Y) 3CL^pro^ S2 pocket were identical to these of α‐CoV and β‐CoV 3CL^pro^s, respectively (Figure 6C). Meanwhile, their substrate preferences at P2 and P4 sites were highly compatible with the characterization of α‐ and β‐CoV 3CL^pro^, respectively (Figure 6E,F). These results suggested that the newly discovered 3CL^pro^s relied on a similar substrate‐binding mode for catalyzing substrates. They represent the ancestral state of the modern 3CL^pro^, of which PsNV and MLeV may be the ancestors of α‐ and β‐CoV, respectively.

### 2.7 The S2 Pocket Dominates the Genus‐Specific Inhibitory Potency of PF‐07321332 on Different CoV 3CL^pro^s

**Figure 7:**
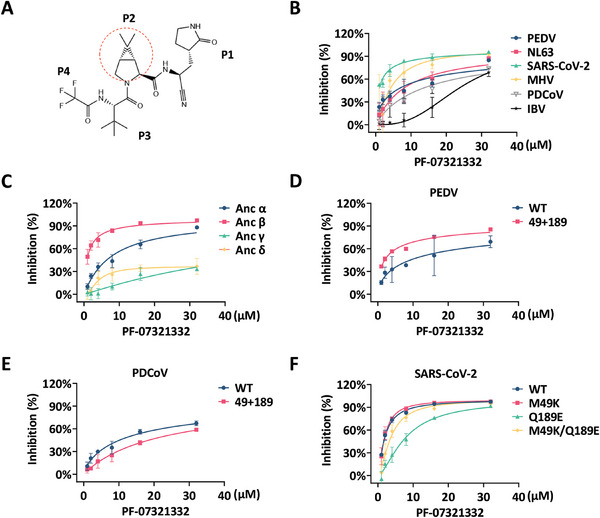
S2 pocket is a pivotal factor for variable inhibitory potency of PF‐07321332 against 3CL^pro^s from different genera. A) Chemical structures of PF‐07321332 P1, P2, P3, and P4 moieties. P1, P2, and P4 moieties correspond to the substrate‐binding pocket in the active site of CoV 3CL^pro^. B) Inhibitory activity profiles of PF‐07321332 against the six CoV 3CL^pro^s. C) Inhibitory activity profiles of PF‐07321332 against the four ancestral 3CL^pro^s. D) Inhibitory activity profiles of PF‐07321332 against PEDV 3CL^pro^ WT or mutant. “49” represents the substitution of 41–54 loop. “189” represents the substitution of 187–190 loop. E) Inhibitory activity profiles of PF‐07321332 against PDCoV 3CL^pro^ WT or mutant. “49” represents the substitution of 41–54 loop. “189” represents the substitution of 187–190 loop. F) Inhibitory activity profiles of PF‐07321332 against SARS‐CoV‐2 3CL^pro^ WT or mutants.

Peptidomimetic 3CL^pro^ inhibitors achieve specific inhibitory efficacy by mimicking the binding mode of CoV 3CL^pro^ with peptide substrates. Therefore, the S2 pocket‐mediated genus‐specific substrate selectivity of CoV 3CL^pro^ serves as a crucial consideration for inhibitor design. The P2 moiety in PF‐07321332 was modified with a dimethyl cyclopropyl proline (DMCP) as a cyclic leucine mimetic (**Figure** **7**A). Given the broad treatment of COVID‐19 and the characteristic of P2 moiety, we explored the connection between the S2 pocket and drug selectivity using PF‐07321332 as a model. Among the six selected 3CL^pro^s, PF‐07321332 displayed a more potent inhibitory effect toward β‐genus SARS‐CoV‐2 and MHV 3CL^pro^s (Figure 7B; and Figure S8A,B and Table S1, Supporting Information). Consistently, PF‐07321332 exhibited the most pronounced potency against Anc β, and the weaker inhibition against Anc γ and δ, suggesting a correlation between the plasticity of S2 pocket and differential drug potency (Figures 7C and S8C and Table S2, Supporting Information).

To further characterize the correlation between S2 pocket and differences in inhibitory potency, the inhibitory effects of PF‐07321332 against PEDV and PDCoV 3CL^pro^ mutants with S2 pocket substitution were determined. Consistent with altered substrate preference at P2 site, PF‐07321332 displayed an approximately threefold enhancement in inhibitory effect against PEDV 49+189 mutant (Figures 7D and S8D and Table S3, Supporting Information). However, no enhanced inhibition effect of PF‐07321332 was observed against the PDCoV 49+189 mutant (Figures 7E and S8E and Table S3, Supporting Information). The S2 pocket in PDCoV 49+189 mutant is more unstable compared to PEDV 49+189 mutant, which could be responsible for the inability to accommodate the bulky DMCP (Figure S3H, Supporting Information). To explore the molecular mechanisms underlying the improved inhibitory effect against PEDV mutant, we performed MD simulations of PEDV WT or 49+189 mutant in complex with PF‐07321332, the S2 pocket in PEDV WT 3CL^pro^ possessed poorer stability in accommodating the large P2 moiety compared to the replaced S2 pocket (Figure S8F, Supporting Information). Moreover, the intermolecular interactions of modified PEDV 3CL^pro^ with PF‐07321332 were identical to that of SARS‐CoV‐2 3CL^pro^ (Figure S8G,H, Supporting Information).^[^ ^19^ ^]^ The alteration in binding mode of S2 pocket resulted in a considerable enhancement in the binding free energy relative to that of PEDV 3CL^pro^ WT (−3.41 kcal mol^−1^), and residue 49 in S2 pocket contributed to a considerable proportion of this increase (−0.95 kcal mol^−1^) (Figure S8I, Supporting Information). Additionally, we investigated the impact of the S2 pocket on another SARS‐CoV‐2 3CL^pro^ inhibitor, Simnotrelvir (Figure S8J,M, Supporting Information). The consistent results from our simulations further highlight the critical role of the S2 pocket in drug selectivity. We further evaluated the inhibitory efficacy of PF‐07321332 on several SARS‐CoV‐2 3CL^pro^ S2 pocket mutants. Mutants Q189E and M49K/Q189E, characterized by the S2 pocket of γ‐ or δ‐CoV 3CL^pro^s, displayed decreased sensitivity to PF‐07321332 (Figures 7F and S8N and Table S4, Supporting Information). These results demonstrated that the S2 pocket is a key factor in the genus‐specific inhibitory effects of PF‐07321332 against different 3CL^pro^s.

## 3 Discussion

In this work, we uncovered the detailed mechanisms for substrate‐binding modes of representative CoV 3CL^pro^s from four genera. Our analyses showed that the substrate‐binding mode in CoV 3CL^pro^ S2 pocket exhibited significant genus‐specific diversity and the substrate selectivity of CoV 3CL^pro^ was largely attributable to the S2 pocket. The newly identified CoV 3CL^pro^s in fish and amphibians have provided insights into the evolutionary history of CoV. Moreover, the differential plasticity of S2 pocket was proved to highly correlate with the inhibitory effect of PF‐07321332.

Degradation of host proteins by proteases involves two steps, specific substrate‐binding and chemical catalysis. CoV 3CL^pro^ relies on the conserved catalytic dyad (H41 and C145) to catalyze the breaking of the peptide bond between P1 and P1′ sites in substrate. The evolutionarily conserved S1 pocket of CoV 3CL^pro^ employs F140, H163, and E166 to anchor the side chain of Gln.^[^ ^20^, ^21^, ^22^ ^]^ In contrast to the absolute requirement for Gln at P1 site, CoV 3CL^pro^ possesses genus‐specific substrate selectivity at P2, P4, and P1′ sites. This suggested that the substrate landscapes of 3CL^pro^ from different genera are variable. We previously validated that porcine STAT2 could be degraded by PDCoV 3CL^pro^ from δ‐genus but not cleaved by PEDV and TGEV 3CL^pro^s from α‐genus, and that human CoV 3CL^pro^s from α‐ and β‐genus including SARS‐CoV, MERS‐CoV, HCoV‐229E, HCoV‐OC43, and HCoV‐NL63 could not cleave human STAT2.^[^ ^11^ ^]^ In the study, we revealed that the genus‐specific salt bridge within S2 pocket determined the specific cleavage of STAT2 by PDCoV 3CL^pro^ (Figure 4). Furthermore, in our previous study, SARS‐CoV and SARS‐CoV‐2 3CL^pro^s displayed a stronger cleavage ability for NEMO compared to PEDV 3CL^pro^. SARS‐CoV and SARS‐CoV‐2 3CL^pro^s not only cleaved the common Q231 site in NEMO, but also recognized the Q205 cleavage motif with a Met at the P2 site.^[^ ^12^, ^23^ ^]^ These results imply that different 3CL^pro^s might possess the specific host target proteins, serving as an entry point for elucidating the differential pathogenic mechanisms of different CoVs.

The previous model of CoV evolution indicated bats were the gene source of α‐ and β‐CoVs and birds were the gene source of γ‐ and δ‐CoVs.^[^ ^24^ ^]^ Based on the evolutionary marker residue 54, α‐CoVs are more closely related to β‐CoVs, while γ‐ and δ‐CoVs belong to the same category. In terms of structural characteristics, the intra‐network within the S2 pocket of α‐ and β‐CoV 3CL^pro^s exhibited greater similarity, characterized by a direct hydrogen bond between Y54 and D187. Likewise, the S2 pockets of γ‐ and δ‐CoV 3CL^pro^s maintained a consistent configuration, with W54 and D187 forming a water bridge. The uniformity further demonstrates the potential of evolutionary markers in classifying and exploring the evolutionary process of CoVs.

The crucial role of 3CL^pro^ in facilitating viral replication highlights the conservatism of its active site. Many mutations in the active site of 3CL^pro^ are lethal for CoV, particularly those that disrupt the stable binding mode in the S1 and S2 pockets. This interprets the evolutionary characterization of the genus‐specific S2 pocket in CoV 3CL^pro^. The functional indispensability of residues 49, 54, and 189 in the ancestral 3CL^pro^ has left an indelible mark on the evolutionary process. This imprint is consistently manifested in new 3CL^pro^s, leading to the genus‐specific S2 pockets.

As aquatic and particularly marine metazoa transcriptomes are well‐sampled, more and more novel nidovirus‐like viruses related to *Coronaviridae*, *Arteriviridae*, and *Abyssoviridae* are discovered. We demonstrated that all of the eight newly characterized CoVs encoded a functional 3CL^pro^ for polyprotein processing. These proteases shared a substrate‐binding mode similar to that of modern 3CL^pro^s. However, different from the genus‐specific divergence of S2 pocket in *Orthocoronavirus* 3CL^pro^, S2 pockets in the newly identified 3CL^pro^s were irregular with the disordered 41–54 loop. These analogous but antique signatures indicated the substrate‐binding mode in the modern 3CL^pro^ might evolve from these newly identified 3CL^pro^s. Of the eight newly discovered CoVs, five (HkudNV, TparNV, SilNV, MalbNV, and PsNV) were found in strictly aquatic animals, and two (MLeV and AmexNV) were in part associated with hosts, such as Ambystoma mexicanum and frogs. These data suggested that it might be useful to consider potential routes of interspecies transmission between marine, freshwater, and terrestrial hosts in future studies of CoVs evolution. The PsNV and MLeV 3CL^pro^s exhibited the same S2 pocket characteristics and substrate preferences as modern α‐ and β‐CoV 3CL^pro^s, respectively, representing the possible ancestral state of the genus‐specific CoV 3CL^pro^. Moreover, the evolutionary marker Y54 in S2 pocket is conserved in all eight newly characterized 3CL^pro^s and is consistent with both α‐ and β‐CoV 3CL^pro^s. These evidences suggest that the ancestors of α‐ and β‐CoVs might have spread from marine host to terrestrial host before γ‐ and δ‐CoVs. Taken together, these evolutionary markers in 3CL^pro^ might enable the order of emergence of each cascade to be further determined. Certainly, more investigations are required in the future to explore the selection pressures in evolutionary history that led to the differentiation of CoV 3CL^pro^.

As temperature rises, the structural fluctuations of proteins become more pronounced. In several studies on protease engineering, researchers have introduced salt bridges in the active site to enhance the protease's thermal stability.^[^ ^25^, ^26^, ^27^ ^]^ The α‐CoVs and β‐CoVs mainly infect mammals. However, γ‐CoVs primarily infect birds, and δ‐CoVs have been found to infect both birds and mammals. Birds clearly tend to have higher body temperatures than mammals, including bats, with a range of 38–44 °C.^[^ ^28^ ^]^ In γ‐ and δ‐CoV 3CL^pro^ S2 pocket, a genus‐specific strong salt bridge formed between residues K49 and E189 in these two loops. This suggests that the stable salt bridge in the S2 pocket of γ‐ and δ‐CoV 3CL^pro^ might be a crucial factor for maintaining enzymatic activity at higher temperatures. Interestingly, the eight newly identified 3CL^pro^ were all derived from poikilothermic animals, with their habitats typically ranging in temperature from 6.3 to 30 °C. Lower environmental temperatures imply that the evolutionary pressure on the thermal stability of these 3CL^pro^s’ active sites is less pronounced, which aligns with the high mutation rates observed in these S2 pockets. Additionally, most research focuses on the potential cross‐species transmission of γ‐ and δ‐CoVs from birds to mammals, with few reports of reverse transmission. This unidirectional transmission might also be associated to differences in thermal stability among 3CL^pro^s, particularly in the S2 pocket.

Thorough elucidation of substrate‐active site interactions is crucial for rational drug design. In the design and optimization of peptidomimetic inhibitors against CoV 3CL^pro^, retention of substrate‐binding mode mainly relies on P1‐P4 moieties in the inhibitors, in addition to the warhead in P1′ moiety. The P1 moiety is frequently substituted for a 5‐membered ring (γ‐lactam) derivative of glutamine. To obtain better specificity, the main chain hydrogen bonds between P3 and E166 have been retained. Therefore, synthetic efforts usually optimize the substituents at P2 and P4 moieties.^[^ ^20^, ^29^, ^30^, ^31^, ^32^ ^]^ Our results demonstrated that the S2 pocket conferred genus‐specific substrate selectivity of 3CL^pro^ and correlated with the inhibitory potency of PF‐07321332. Compared to the 3CL^pro^ from other three genera, β‐CoV 3CL^pro^ S2 pocket exhibited higher phenotypic plasticity and broader tolerance. Hence, the introduction of a large moiety at P2 site to generate sufficient binding enthalpy may be an effective avenue to optimize inhibitors against β‐CoV. Due to the correlation between S2 and S4 pockets, α‐CoV 3CL^pro^ holds a lower tolerance for large groups at P2 and P4 sites, which means that the size of P2 and P4 moieties may need to be taken into account when designing specific inhibitors for α‐CoV. Previously, very little attention has been given to the design of drugs against γ‐ and δ‐CoV. However, three Haitian children with acute undifferentiated febrile illness tested positive for PDCoV recently, highlighting the risk of PDCoV cross‐species transmission and its potential threat to public health. The stability of the salt bridge between K49 and E189 is essential for substrate binding in γ‐ and δ‐CoV 3CL^pro^, leading to a more restricted selectivity at P2 site. This suggests that the P2 substituent should be small enough to retain the salt bridge in S2 pocket when designing specific inhibitors against γ‐ and δ‐CoV 3CL^pro^. Certainly, given the design of broad‐spectrum inhibitors of CoV 3CL^pro^, γ‐ and δ‐CoV 3CL^pro^, which possess the most limited substrate selectivity at P2 site, may be a suitable template.

The 41–54 and 187–190 loops of CoV 3CL^pro^ S2 pocket contributed to the differential inhibitory effect of PF‐07321332, suggesting that CoV 3CL^pro^ could gain drug resistance via changes at these loops, as substitutions in these loops would reduce the number of inhibitor/enzyme interactions while the binding of the substrate is maintained. Moreover, the distinct sensitivity of PEDV and PDCoV 49+189 mutants toward PF‐07321332 underscored the potential role of S2 pocket destabilization as a driving force behind acquired resistance. In the Global Initiative on Sharing All Influenza Data (GISAID) (https://gisaid.org/hcov19‐variants/),^[^ ^33^ ^]^ frequent mutations have been documented within the 41–54 loop region of the S2 pocket, such as S46F, E47N/K, L50F, and the latest addition, T45N, indicating that these mutations were present in circulating SARS‐CoV‐2 strains. Notably, a L50F 3CL^pro^ substitution in SARS‐CoV‐2 3CL^pro^ was recently identified in the individuals treated with PF‐07321332 and was reported to accelerate resistance selection,^[^ ^34^ ^]^ further emphasizing the important role of these two loops and rationalizing the origin of resistance mechanisms to treatment with 3CL^pro^ inhibitors.

## 4 Experimental Section

### Plasmids

The cDNA expression constructs encoding PEDV, NL63, SARS‐CoV‐2, MHV, IBV, and PDCoV 3CL^pro^s were PCR‐amplified and cloned into the C‐terminal hemagglutinin (HA)‐tag‐encoding pCAGGS‐HA‐C plasmid. The DNA fragments encoding the newly identified 3CL^pro^s were synthetized by Tsingke (China) and cloned into the pCAGGS‐HA‐C vector. Mutagenesis of 3CL^pro^ was carried out by overlapping extension PCR using specific mutagenic primers. The construction strategy and effectiveness of the luciferase‐based biosensor plasmids to monitor the activity of CoV 3CL^pro^ have been previously demonstrated in previous studies.^[^ ^35^, ^36^, ^37^, ^38^, ^39^, ^40^ ^]^ To maintain consistency with the peptide substrate in the complex crystal structures (PDB Entry: 4ZUH and 7N89), the nsp4/nsp5 autocleavage sequence (TSAVLQ↓SGFRKM) of SARS‐CoV‐2 3CL^pro^ was fused to luciferase reporter plasmids to monitor the activity of CoV 3CL^pro^ (233DS). A luciferase reporter plasmid that contained oligonucleotides corresponding to ENLYFQ↓YS, a cleavage motif by the tobacco etch virus (TEV) 3C^pro^, was used as the reporter control.^[^ ^41^ ^]^ A positional scanning peptide library was constructed by saturation mutagenesis at P2, P4, and P1′ sites on the nsp4/nsp5 autocleavage sequence for a total of 19 × 3 reporter systems. All the constructs were validated by DNA sequencing. Details of the abbreviations used for different mutations are provided in Table S5 (Supporting Information).

### Luciferase Reporter Gene Assays

Human embryonic kidney (HEK‐293T) cells, obtained from the China Center for Type Culture Collection, were cultured at 37 °C in 5% CO_2_ in Dulbecco's modified Eagle's medium (DMEM) supplemented with 10% fetal bovine serum. Luciferase reporter constructs and their controls were used to detect 3CL^pro^ activity. HEK‐293T cells plated on 24‐well plates were transfected with various 3CL^pro^ expression plasmids or empty control plasmids, together with the luciferase reporter plasmid and pRL‐TK (Promega), which was used as an internal control to normalize the transfection efficiency. At 18 h post‐transfection, the cells were lysed, and a luciferase reporter assay system (Promega) was utilized to determine the luciferase activity in the lysed cells. The activities were normalized to the corresponding Renilla luciferase activity. The relative cleavage activities of 3CL^pro^ in the saturation mutagenesis experiment represent the ratio of the fold induction on the point mutant substrate to that of the WT substrate (the nsp4/nsp5 autocleavage sequence).

### Western Blotting Analysis

After determining the amount of luciferase activity in the lysed cells, HEK‐293T cells in 24‐well plates were harvested. The samples were resolved by SDS‐PAGE and then transferred to PVDF membranes (Millipore Sigma, Burlington, MA) to determine the protein expression levels. The membranes were then incubated with primary and secondary antibodies. The overexpression of 3CL^pro^s was evaluated using an anti‐HA antibody (Medical and Biological Laboratories, Nagoya, Japan). An antigoat monoclonal secondary antibody (Promega) was used to analyze the expression level of each luciferase reporter gene. An anti‐β‐actin mouse monoclonal antibody (Beyotime, Shanghai, China) was utilized to monitor β‐actin's expression level to confirm that protein loading was equal for all samples.

### Homology Modeling

The crystal structures of PEDV, NL63, SARS‐CoV‐2, MHV, IBV, and PDCoV 3CL^pro^s were obtained from the Protein Data Bank (PDB IDs 4ZUH, 3TLO, 7N89, 6JIJ, 2Q6D, and 7WKU, respectively). The crystal structure of SARS‐CoV‐2 3CL^pro^ in complex with the octapeptide Ac‐SAVLQSGF‐CONH2, corresponding to the nsp4/nsp5 autocleavage site of SARS‐CoV‐2, was obtained from Protein Data Bank (PDB ID 7N89).^[^ ^42^ ^]^ The remaining five complexes were generated by superposition with this crystal complex based on the high homology. The structural models of four ancestral 3CL^pro^ Anc α, Anc β, Anc γ, and Anc δ were generated using ColabFold.^[^ ^43^ ^]^ The structures of PEDV and PDCoV 49+189 mutants were obtained using the crystal structure of PEDV/PDCoV and SARS‐CoV‐2 3CL^pro^ as multiple templates.^[^ ^44^ ^]^ The structures of PEDV WT and PEDV 49+189 mutant in complex with PF‐07321332 were generated using the crystal structure of SARS‐CoV‐2 3CL^pro^ with PF‐07321332 (PDB ID 7RFS). The octapeptide Ac‐SAVLQSGF‐CONH2 in the crystal structure of SARS‐CoV‐2 3CL^pro^ (PDB ID 7N89) was complemented to the dodecapeptide while retaining the natural N‐ and C‐terminals using software in the SYBYL‐X program (v.2.0; https://omictools.com/sybyl‐x‐tool). Mutations in 3CL^pro^s or the substrate were constructed with SYBYL‐X.

### MD Simulation

MD simulations were conducted using the Amber ff14SB force field (with the TIP3P water model) implemented in the GROMACS 2018 software package, as described before. For each system, MD simulations were collected for 150 ns and repeated three times.^[^ ^45^, ^46^ ^]^ Each complex was inserted into and centralized in a triclinic box (box dimensions: 9 nm × 12 nm × 9 nm). Molecular systems were neutralized via the addition of a certain number of the counterions sodium (Na+) or chloride (Cl−), and NaCl at 150 mM was used to mimic physiological conditions. For energy minimization and relaxation, each system was energy minimized for 3000 steps using a conjugate gradient algorithm. Following minimization, each system was gradually heated from 0 to 310 K in 500 ps, then equilibrated at that temperature for another 500 ps. The standard temperature was kept constant at 310 K, then equilibration was performed under constant pressure for 150 ns with no position restrictions on the protein. During MD simulation, the SHAKE algorithm was applied to constrain all bonds involving hydrogen atoms. The temperature was controlled using a modified Berendsen thermostat (V‐rescale algorithm) with a collision frequency of 0.2 ps. The pressure was maintained at 1 bar using a Parrinello–Rahman barostat with a compressibility of 4.5 × 10^−5^ bar^−1^ and a coupling constant of 2.0 ps. The Particle Mesh Ewald method was utilized to treat long‐range electrostatic interactions. A 2 fs step was applied to calculate the motion equations using the Leap‐Frog integrator for equilibration steps. The coordinates of the atoms were taken every 10 ps and used for the final analysis. The RMSF values, vdW interactions, and electrostatic interactions were calculated using the GROMACS package. Atomic charges of PF‐07321332 were obtained using the restrained electrostatic potential (RESP) method at the B3LYP/6‐311G** level. A total of 41 molecular assemblies were simulated over an aggregate time of ≈18 µs (details are provided in Figures S9 and S10 and Table S6, Supporting Information).

### MM‐PBSA Method

The MM‐PBSA method^[^ ^47^ ^]^ was used to calculate the binding affinity of PF‐07321332 to CoV 3CL^pro^. For each simulated system, 1000 snapshots were extracted from the last 100 ns of the MD trajectory at intervals of 100 ps for calculations. In the MM‐PBSA scheme, the binding free energy (Δ*G*) was computed using the following Equation (1)

(1) $$\Delta G=\Delta E_{\mathrm{MM}}+\Delta G_{\mathrm{sol}}$$

In Equation (1) *E*_MM_ defines the interaction energy between the protein and the ligand, as calculated by molecular mechanics in the gas phase. Δ*G*_sol_ is the desolvation free energy for transferring the ligand from water to the calculated binding area using Poisson–Boltzmann (PB) equations. The terms for each complex Δ*E*_MM_ and Δ*G*_sol_ are calculated using Equations (2) and (3)

(2) $$\Delta E_{\mathrm{MM}}=\Delta E_{\mathrm{ele}}+\Delta E_{\mathrm{vdW}}$$

(3) $$\Delta G_{\mathrm{sol}}=\Delta G_{P}+\Delta G_{\mathrm{NP}}$$

In Equation (2) Δ*E*_ele_ and Δ*E*_vdW_ represent the electrostatic and vdW interactions in the gas phase, respectively. In Equation (3) Δ*G*_P_ is the electrostatic or polar contribution to the free energy of solvation, and the term Δ*G*_NP_ is the nonpolar or hydrophobic contribution to the solvation free energy.

### Cross‐Correlation Analysis

The cross‐correlation matrix elements C*_ij_*, which reflect fluctuations in the coordinates of the Ca atoms relative to their mean positions, were calculated from the last 100 ns of the MD trajectory for each system using the following equation, where the angle brackets represent the mean times over the recorded snapshots

(4) $$C_{\mathrm{ij}}=\frac{\langle \Delta r_{i}\cdot \Delta r_{j}\rangle }{{\left(\langle \Delta r_{i}^{2}\rangle \cdot \langle \Delta r_{j}^{2}\rangle \right)}^{2}}$$

Δr*_i_* indicates the displacement vector from the mean position for the *i*th residue. The value of C*_ij_* ranges from −1 to 1. A positive C*_ij_* value represents a correlated motion of C_α_ atoms of the *i*th and *j*th residues, while a negative value of C*_ij_* describes an anticorrelated motion.

### Ancestral Sequence Reconstruction

Based on the *Orthocoronavirinae* subfamily in ICTV, 44 representative CoVs species were selected. The 44 CoV 3CL^pro^ amino acid sequences were aligned using Muscle (version 3.8.31), and the alignment was corrected manually. The best substitution model for analysis of amino acid sequences of 3CL^pro^ was selected based on the lowest Bayesian information criterion (BIC) score in MEGA‐X 10.1.8.^[^ ^48^ ^]^ The amino acid substitution model LG+F+G+I (gamma distribution with invariant sites) was used to generate phylogenetic trees.^[^ ^49^ ^]^ The maximum‐likelihood phylogenetic trees were statistically validated using 1000 bootstrap replicates.

To infer the ancestral amino acid sequences of CoV 3CL^pro^, the phylogeny of the *Coronaviridae* family was constructed using the same strategy described above. MLeV 3CL^pro^ from the *Letovirinae* subfamily was used as the outgroup to root the tree. The ancestral sequences from the phylogenetic nodes corresponding to Anc α, Anc β, Anc γ, and Anc δ were inferred by the maximum‐likelihood method in MEGA‐X 10.1.8 based on amino acid substitutions identified in the LG+F+G+I model.^[^ ^50^ ^]^

### Hydrogen Bond Calculations

The percentage of time that a hydrogen bond existed during a trajectory was calculated using the HBonds Plugin from Visual Molecular Dynamics^[^ ^51^ ^]^ and averaged over 300 ns (i.e., the last three 100 ns of each repetition system). A hydrogen bond was defined as having a donor–acceptor distance of a maximum of 3.5 Å, where only the polar atoms (nitrogen, oxygen, sulfur, and fluorine) were involved. The donor‐hydrogen acceptor angle was defined as being < 40°. Hydrogen bonds were summed over each residue and substrate, except when otherwise indicated.

### Microassembly of CoV‐Aligned Reads

We employed Trimmomatic (version 0.39) to perform quality control on FASTQ files, using parameters SLIDINGWINDOW:5:20, LEADING:5, TRAILING:5, and MINLEN:50 to filter out low‐quality reads. The resulting filtered fastq data were then subjected to assembly using Coronaspades (version 3.15.3), specifically designed for RNA virus data, yielding scaffold results. Additionally, VirSorter2 (version 2.2.3) was utilized to extract complete RNA virus sequences from the assembly data, with parameters set as follows: −seqname‐suffix‐off, −viral‐gene‐enrich‐off, −prep‐for‐dramv, −include‐groups RNA, and −min‐score 0.5.

Following the acquisition of the filtered scaffold fasta data, the BLAST software was employed to identify sequences most closely related to the 3CL^pro^ sequence. Initially, the makeblastdb command with the fasta sequences obtained from VirSorter to create a corresponding sequence database was utilized. Subsequently, the TBLASTN command was executed to align the nsp.faa sequence to the created nucleotide database, using parameters ‐outfmt 7 and ‐evalue 10. From the BLAST results, sequences corresponding to e‐values less than 10^^‐10^ were selected, and the relevant sequences were extracted from the corresponding fasta files based on the hit's start and end positions.

### Inhibition Assays

PF‐07321332 (Selleck, China) was suspended in DMSO at a concentration of 1 mg mL^−1^ following the manufacturer's instructions. Stock aliquots were stored at −80 °C. For each experiment, the compounds were diluted to the desired concentration with DMEM supplemented with 10% FBS. To more realistically compare the differences in inhibitory effect of PF‐07321332 against CoV 3CL^pro^s, a high‐throughput luciferase‐based biosensor with the nsp4/nsp5 autocleavage site (TSAVLQ↓SGFRKM) was used to determine the inhibitory potency of PF‐07321332 against CoV 3CL^pro^s in the cellular environment. HEK‐293T cells plated in 24‐well plates were transfected with 3CL^pro^ expression plasmids, together with the luciferase reporter plasmid and pRL‐TK. At 6 h post‐transfection, the cells were treated with different concentrations of PF‐07321332 (1, 2, 4, 8, 16, or 32 µm) diluted with DMEM. After 12 h, the cells were lysed, and a luciferase reporter assay system was utilized to determine luciferase activity in the lysed cells. The inhibitory potency represents the ratio of the fold induction in the EXPERIMENTAL group (with PF‐07321332) to the control group (with DMSO).

### Statistical Analysis

Statistical analyses were performed using Prism v8.0 (GraphPad Software). All results for statistical analysis are presented as the means ± SD of at three experiments. Significant differences were detected using Student's t‐test. Statistical significance is denoted as *P < 0.05, **P < 0.01, ***P < 0.001, ****P < 0.0001 or NS (not significant, P > 0.05).

## Conflict of Interest

The authors declare no competing interests.

## Author Contributions

J.W.Z. and S.B.X. designed the experiments. J.W.Z., P.S., Z.X.Y., and Z.L. performed the experiments. J.W.Z., P.S., T.Q.W., J.H.G., R.H.Q., and J.S.Z. conducted the bioinformatics analysis. J.W.Z., D.G.W., G.Q.P., S.B.X., and L.R.F. wrote the paper. S.B.X. and L.R.F. supervised the research, coordination and strategy.

## Supporting information



Supporting Information

## Data Availability

The data that support the findings of this study are available from the corresponding author upon reasonable request.
