# Supplementary material for: The S2 Pocket Governs the Genus‐Specific Substrate Selectivity of Coronavirus 3C‐Like Protease
Source: Adv Sci (Weinh). 2024 Oct 8;11(44):2407766. doi: 10.1002/advs.202407766 (PMC11600255; doi:10.1002/advs.202407766)
Supplement: Supplementary file 1 — Supporting Information [file ADVS-11-2407766-s001.docx]

**The S2 pocket governs the genus-specific** **substrate selectivity of coronavirus 3C-like protease**

Junwei Zhou^1,2^, Peng Sun^1,2^, Zhixiang Yang^1,2^, Taiquan Wang^1^, Jiahui Guo^1,2^, Runhui Qiu^1,2^, Zhuang Li^1,2^, Dengguo Wei^1,2^, Jinshui Zheng^1^, Guiqing Peng^1,2^, Liurong Fang^1,2*^, Shaobo Xiao^1,2*^

^1^State Key Laboratory of Agricultural Microbiology, College of Veterinary Medicine, Huazhong Agricultural University, Wuhan 430070, China

^2^The Key Laboratory of Preventive Veterinary Medicine in Hubei Province, Cooperative Innovation Center for Sustainable Pig Production, Wuhan 430070, China

*Correspondence: College of Veterinary Medicine, Huazhong Agricultural University, 1 Shi-zi-shan Street, Wuhan 430070, Hubei, PR China. E-mail: [vet@mail.hzau.edu.cn](mailto:vet@mail.hzau.edu.cn) (S.X.) and [fanglr@mail.hzau.edu.cn](mailto:fanglr@mail.hzau.edu.cn) (L.F.).

**Supplemental Materials**

**Tables S1-S4.** Values obtained for the luciferase-based inhibition assays.

**Tables S5.** Abbreviations for different mutations.

**Tables S6.** Detail of the molecular assemblies for MD simulations in this study

**Figure S1.** Details of the substrate-binding mode in CoV 3CL^pro^s.

**Figure S2.** Simulations of six 3CL^pro^s in complex with P2-M substitution substrate**.**

**Figure S3.** Genus-specific S2 pocket modulates the substrate preference of CoV 3CL^pro^ at P2 site.

**Figure S4.** The variation of the hydrogen bond occupancy in P3, P1 and P2′ sites for P4-P substitution system relative to the WT system.

**Figure S5.** Residue 49 in the S2 pocket govern the capacity of 3CL^pro^ to recognize and cleave P1′-E substrate.

**Figure S6.** The four ancestral 3CL^pro^s possess the conserved and specific substrate-binding mode.

**Figure S7.** The newly discovered 3CL^pro^s exhibit a substrate-binding mode similar to that of modern 3CL^pro^s.

**Figure S8.** The specific S2 pocket is responsible for the more pronounced inhibitory effect of PF-07321332 towards β-CoV 3CL^pro^.

**Figure S9.** Plot of the Root Mean Square Deviation (RMSD) values

**Figure S10.** Distance distributions between residue 49/189 and P2-Leu in SARS-CoV-2 nsp4/5 substrate systems.

**Table S1. IC_50_ values obtained for the luciferase-based inhibition assays.**

| **Protease** | **IC_50_(μM)** |
| --- | --- |
| PEDV | 7.63 |
| NL63 | 8.612 |
| SARS-CoV-2 | 1.12 |
| MHV | 4.463 |
| IBV | 24.28 |
| PDCoV | 14.59 |

**Table S2. IC_50_ values obtained for the luciferase-based inhibition assays.**

| **Protease** | **IC_50_(μM)** |
| --- | --- |
| Anc α | 7.775 |
| Anc β | 1.068 |
| Anc γ | 54.92 |
| Anc δ | ~ |

**Table S3. IC_50_ values obtained for the luciferase-based inhibition assays.**

| **Protease** | **IC_50_(μM)** |
| --- | --- |
| PEDV | 12.53 |
| PEDV+49+189 | 2.685 |
| PDCoV | 13.10 |
| PDCoV+49+189 | 22.70 |

**Table S4. IC_50_ values obtained for the luciferase-based inhibition assays.**

| **Protease** | **IC_50_(μM)** |
| --- | --- |
| SARS-CoV-2 | 1.938 |
| SARS-CoV-2-M49K | 1.872 |
| SARS-CoV-2-Q189E | 7.854 |
| SARS-CoV-2-M49K/Q189E | 3.634 |

**Table S5.** **Abbreviations for different mutations.**

| Mutant systems | Abbreviations |
| --- | --- |
| PEDV 3CL^pro^ in complex with P2-M substitution substrate | PEDV-P2-M |
| NL63 3CL^pro^ in complex with P2-M substitution substrate | NL63-P2-M |
| SARS-CoV-2 3CL^pro^ in complex with P2-M substitution substrate | SARS-CoV-2-P2-M |
| MHV 3CL^pro^ in complex with P2-M substitution substrate | MHV-P2-M |
| IBV 3CL^pro^ in complex with P2-M substitution substrate | IBV-P2-M |
| PDCoV 3CL^pro^ in complex with P2-M substitution substrate | PDCoV-P2-M |
| PEDV 3CL^pro^ in complex with P4-P substitution substrate | PEDV-P4-P |
| NL63 3CL^pro^ in complex with P4-P substitution substrate | NL63-P4-P |
| IBV 3CL^pro^ in complex with P1′-E substitution substrate | IBV-P1′-E |
| PDCoV 3CL^pro^ in complex with P1′-E substitution substrate | PDCoV-P1′-E |
| PEDV 3CL^pro^ with the substitution of 41-54 loops | PEDV 49 |
| PEDV 3CL^pro^ with the substitution of 187-190 loops | PEDV 189 |
| PEDV 3CL^pro^ with the substitution of 41-54 and 187-190 loops | PEDV 49+189 |
| PDCoV 3CL^pro^ with the substitution of 41-54 loops | PDCoV 49 |
| PDCoV 3CL^pro^ with the substitution of 187-190 loops | PDCoV 189 |
| PDCoV 3CL^pro^ with the substitution of 41-54 and 187-190 loops | PDCoV 49+189 |

**Table S6.** **Detail of the molecular assemblies for MD simulations in this study**

| Complex systems | Simulation  time (ns) | Runs^a^ |
| --- | --- | --- |
| PEDV 3CL^pro^ in complex with the nsp4/nsp5 auto-cleavage site of SARS-CoV-2 | 150 | 3 |
| NL63 3CL^pro^ in complex with the nsp4/nsp5 auto-cleavage site of SARS-CoV-2 | 150 | 3 |
| SARS-CoV-2 3CL^pro^ in complex with the nsp4/nsp5 auto-cleavage site of SARS-CoV-2 | 150 | 3 |
| MHV 3CL^pro^ in complex with the nsp4/nsp5 auto-cleavage site of SARS-CoV-2 | 150 | 3 |
| IBV 3CL^pro^ in complex with the nsp4/nsp5 auto-cleavage site of SARS-CoV-2 | 150 | 3 |
| PDCoV 3CL^pro^ in complex with the nsp4/nsp5 auto-cleavage site of SARS-CoV-2 | 150 | 3 |
| PEDV 3CL^pro^ in complex with P2-M substitution substrate | 150 | 3 |
| NL63 3CL^pro^ in complex with P2-M substitution substrate | 150 | 3 |
| SARS-CoV-2 3CL^pro^ in complex with P2-M substitution substrate | 150 | 3 |
| MHV 3CL^pro^ in complex with P2-M substitution substrate | 150 | 3 |
| IBV 3CL^pro^ in complex with P2-M substitution substrate | 150 | 3 |
| PDCoV 3CL^pro^ in complex with P2-M substitution substrate | 150 | 3 |
| PEDV 3CL^pro^ in complex with P4-P substitution substrate | 150 | 3 |
| NL63 3CL^pro^ in complex with P4-P substitution substrate | 150 | 3 |
| SARS-CoV-2 3CL^pro^ in complex with P4-P substitution substrate | 150 | 3 |
| MHV 3CL^pro^ in complex with P4-P substitution substrate | 150 | 3 |
| IBV 3CL^pro^ in complex with P4-P substitution substrate | 150 | 3 |
| PDCoV 3CL^pro^ in complex with P4-P substitution substrate | 150 | 3 |
| PEDV 3CL^pro^ in complex with P1′-E substitution substrate | 150 | 3 |
| NL63 3CL^pro^ in complex with P1′-E substitution substrate | 150 | 3 |
| SARS-CoV-2 3CL^pro^ in complex with P1′-E substitution substrate | 150 | 3 |
| MHV 3CL^pro^ in complex with P1′-E substitution substrate | 150 | 3 |
| IBV 3CL^pro^ in complex with P1′-E substitution substrate | 150 | 3 |
| PDCoV 3CL^pro^ in complex with P1′-E substitution substrate | 150 | 3 |
| SARS-CoV-2 T25S/M49K/Q189E mutant in complex with P1′-E substitution substrate | 150 | 3 |
| PEDV 3CL^pro^ in complex with PF-07321332 | 150 | 3 |
| PEDV double-loop substitution mutant in complex with PF-07321332 | 150 | 3 |
| Anc α in complex with the nsp4/nsp5 auto-cleavage site of SARS-CoV-2 | 150 | 3 |
| Anc β in complex with the nsp4/nsp5 auto-cleavage site of SARS-CoV-2 | 150 | 3 |
| Anc γ in complex with the nsp4/nsp5 auto-cleavage site of SARS-CoV-2 | 150 | 3 |
| Anc δ in complex with the nsp4/nsp5 auto-cleavage site of SARS-CoV-2 | 150 | 3 |
| PEDV 3CL^pro^ with the substitution of 41-54 and 187-190 loop | 150 | 3 |
| PDCoV 3CL^pro^ with the substitution of 41-54 and 187-190 loop | 150 | 3 |
| PEDV 3CL^pro^ in complex with NEMO substrate | 150 | 1 |
| NL63 3CL^pro^ in complex with NEMO substrate | 150 | 1 |
| SARS-CoV-2 3CL^pro^ in complex with NEMO substrate | 150 | 1 |
| MHV 3CL^pro^ in complex with NEMO substrate | 150 | 1 |
| IBV 3CL^pro^ in complex with NEMO substrate | 150 | 1 |
| PDCoV 3CL^pro^ in complex with NEMO substrate | 150 | 1 |
| PEDV 3CL^pro^ in complex with Simnotrelvir | 150 | 3 |
| PEDV double-loop substitution mutant in complex with Simnotrelvir | 150 | 3 |

^a^ Independent MD runs.

**Figure S1**

**Figure S1.** Details of the substrate-binding mode in CoV 3CL^pro^s. **A)** The sequence alignment of the loop at residues 40-54 of 3CL^pro^ in 44 representative CoVs species. The virus 3CL^pro^s used in this study and their GenBank accession numbers are as follows: TGEV, Transmissible gastroenteritis virus (CAB91143.1); MCoV WD1127, Mink coronavirus strain WD1127 (ADI80512.1); HCoV 229E, Human coronavirus 229E (AAG48591.1); NL63, Human coronavirus NL63 (AAS58176.2); BtKYNL63, NL63-related bat coronavirus (APD51481.1); PEDV, Porcine epidemic diarrhea virus (AAK38661.1); ScBatCoV 512, Scotophilus bat coronavirus 512 (ABG47077.1); BtCoVHKU10, Rousettus bat coronavirus HKU10 (AFU92103.1); BtRfAlphaCoV, BtRf-AlphaCoV/HuB2013 (AIA62251.1); MiBatCoV HKU8, Miniopterus bat coronavirus HKU8 (ACA52170.1); MiBatCoV HKU8, Miniopterus bat coronavirus 1 (ACA52163.1); BtCoV, Bat coronavirus CDPHE15/USA/2006 (AGT21332.1); BtNvAlphaCoV, BtNv-AlphaCoV/SC2013 (AIA62264.1); PKBatCoV, Alphacoronavirus Bat-CoV/P.kuhlii/Italy/3398-19/2015 (AZF86123.1); BtMrAlphaCoV, BtMr-AlphaCoV/SAX2011 (AIA62245.1); RhBatCoV HKU2, Rhinolophus bat coronavirus HKU2 (ABQ57207.1); LRNV, Lucheng Rn rat coronavirus (AID16672.2); SmCoV, Wencheng Sm shrew coronavirus (ASF90443.1); SARS-CoV-2, Severe acute respiratory syndrome coronavirus 2 (YP_009724389.1); Bat HpBetaCoV, Bat Hp-betacoronavirus/Zhejiang2013 (AIL94214.1); EiBatCoV, Bat coronavirus (AWV67038.1); RoBatCoV, Rousettus bat coronavirus (AOG30821.1); RoBatCoV HKU9, Rousettus bat coronavirus HKU9 (ABN10910.1); TyBatCoVHKU4, Tylonycteris bat coronavirus HKU4 (ABN10838.1); PiBatCoV HKU5, Pipistrellus bat coronavirus HKU5 (ABN10874.1); MERSCoV, Middle East respiratory syndrome-related coronavirus (AFS88944.1); EriCoV, Betacoronavirus Erinaceus/VMC/DEU/2012 (AGT28262.1); HCoV HKU1, Human coronavirus HKU1 (AAT98578.1); MHV, Murine hepatitis virus (AAU06353.1); HCoV OC43, Human coronavirus OC43 (AAT84351.1); MrufCoV, Rodent coronavirus (ATP66742.1); ChRCoV HKU24, Betacoronavirus HKU24 (AJA91194.1); IBV, Infectious bronchitis virus (AAA70233.1); ACoV, Avian coronavirus (UNG30117.1); DuCoV, Duck coronavirus (AKF17722.1); BcanCoV, Canada goose coronavirus (QCB65096.1); BWCoV SW1, Beluga whale coronavirus SW1 (ABW87819.1); WiCoV HKU20, Wigeon coronavirus HKU20 (AFD29233.1); BulCV HKU11A, Bulbul coronavirus HKU11-934 (ACJ12034.1); WECoV HKU16, White-eye coronavirus HKU16 (AFD29200.1); PDCoV, Porcine coronavirus (AFD29193.2); MunCV HKU13, Munia coronavirus HKU13-3514 (ACJ12061.1); CMCoV HKU21, Common moorhen coronavirus HKU21 (AFD29243.1); NHCoV HKU19, Night heron coronavirus HKU19 (AFD29225.1). **B)** The hydrogen bonding network and occupancy between 3CL^pro^ and P4, P3, P1 and P1′ sites. Hydrogen bond interactions are shown as yellow dashed lines. The red dashed circle represents the conserved oxyanion hole, which is critical for cleavage. Considering the consistency of the binding modes, the schematics are shown using SARS-CoV-2 3CL^pro^ as the template. **C)** Diagram of the three distinct substrate-binding modes in the six CoV 3CL^pro^ S2 pockets in complex with NEMO substrate (aa_226‐237_). Hydrogen bond interactions are shown as yellow dashed lines. Percentage represents the hydrogen bond occupancy between P2 site and residue 49 or 189. **D)** Amino acid sequence logos of the substrates were created using PSSMSearch (http://slim.icr.ac.uk/pssmsearch/), and the height of the letters represents the relative frequency of the amino acid.

**Figure S2**

**A**

**B**

**C**


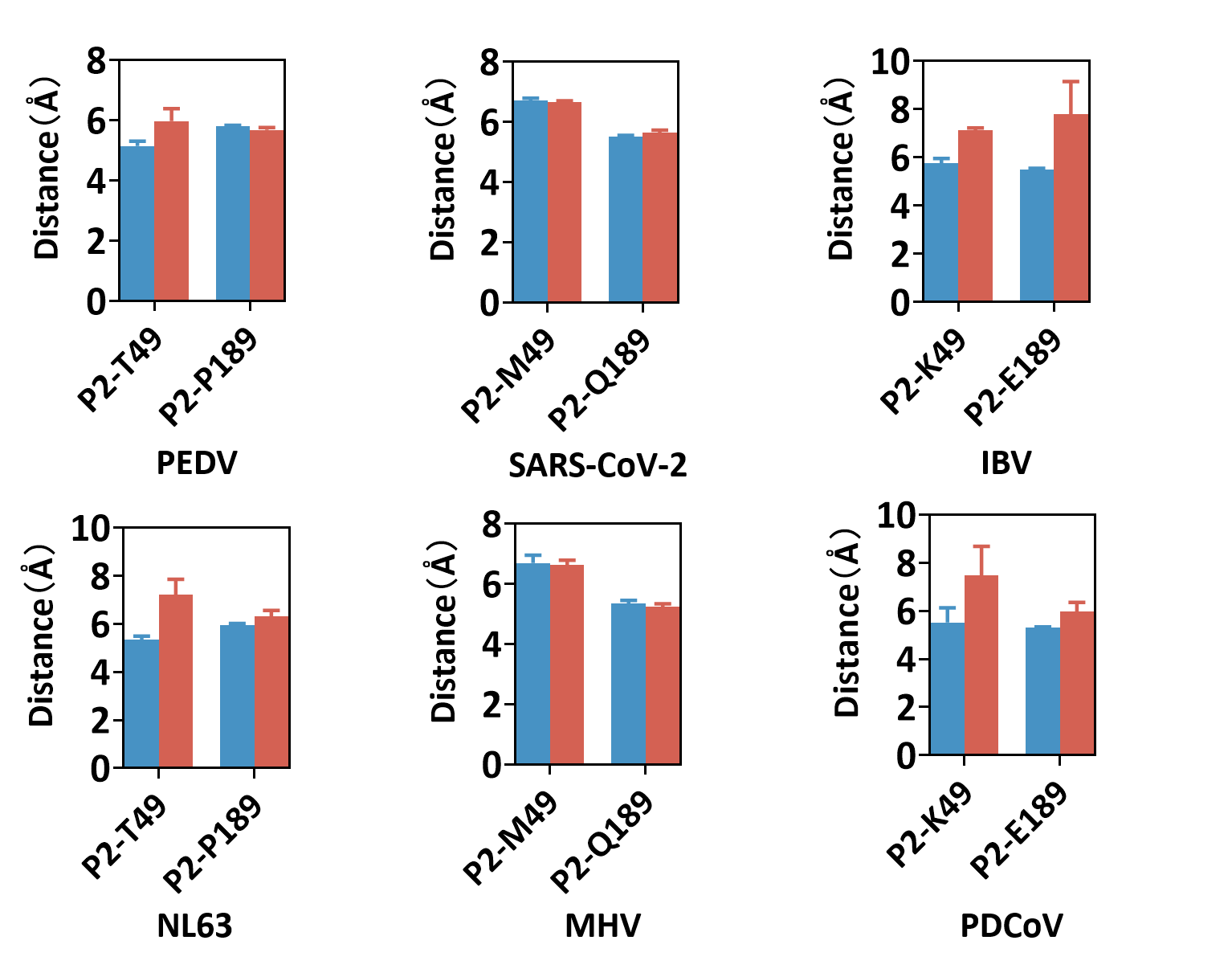


**
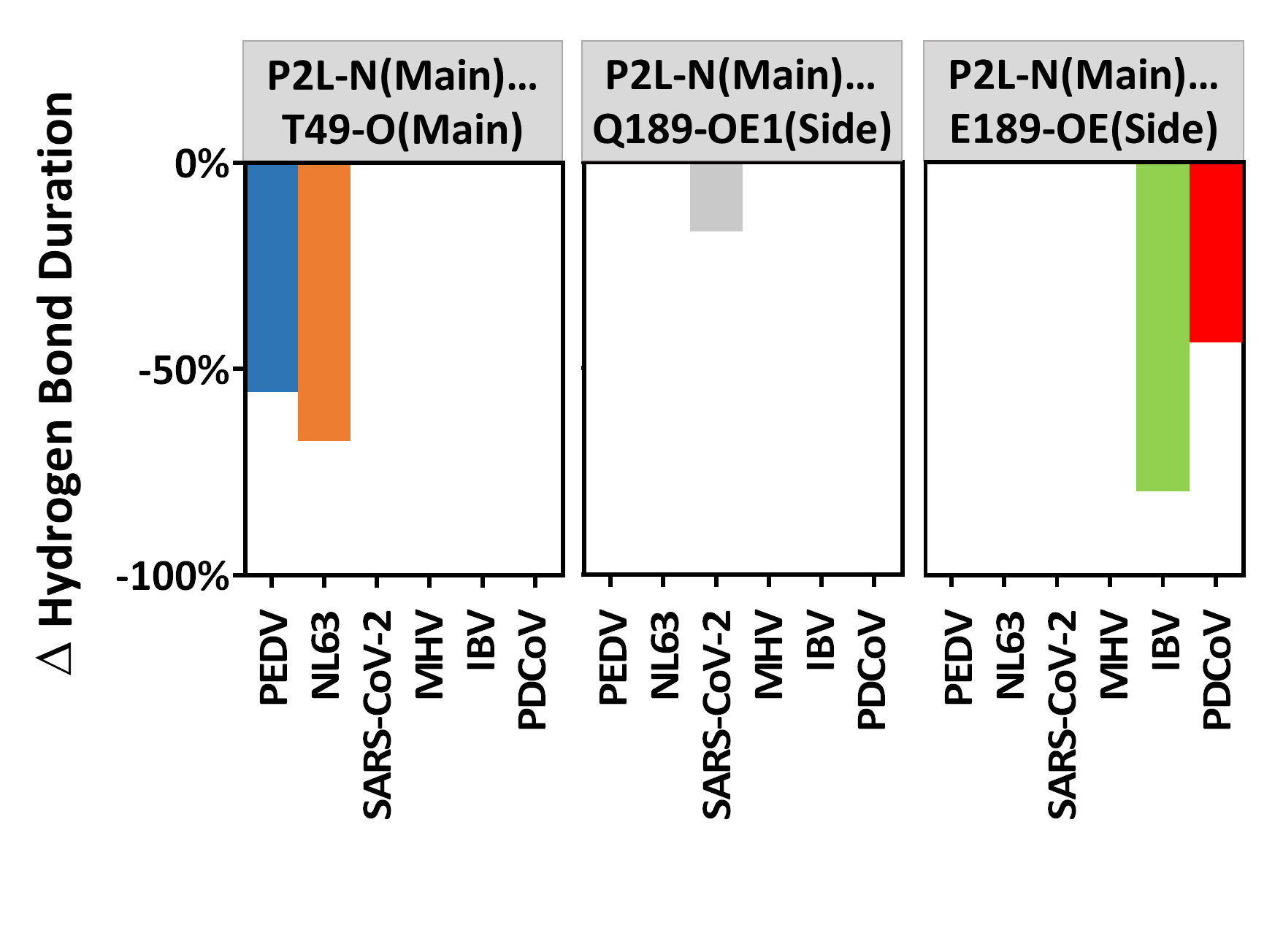
D**

**Figure S2.** Simulations of six 3CL^pro^s in complex with P2-M substitution substrate**. A)** Distance distributions between residue 49 and P2 site in WT (blue) and P2-Met (red) system. Each system possesses three replicates. **B)** Distance distributions between residue 189 and P2 site in WT (blue) and P2-Met (red) system. Each system possesses three replicates. **C)** Time-averaged distance between residue 49 or 189 and P2 site in the WT and P2-Met systems. Blue and red colors represent WT and P2-Met systems respectively. **D)** Changes in hydrogen bond occupancy in S2 pocket for P2-M substitution system relative to the WT system.

**Figure S3**

**Figure S3.** Genus-specific S2 pocket modulates the substrate preference of CoV 3CL^pro^ at P2 site. **A)** Protease activity of 3CL^pro^ X49M/X189Q double-point mutants. αHA, antihemagglutinin; IB, immunoblotting. In comparison to the IBV wild type, the activity of IBV K49M/E189Q mutant was markedly decreased, and therefore, it was not chosen for subsequent experiments. **B-D)** Relative protease activity of 3CL^pro^ X49M/X189Q mutants against P2-V **(B)**, I **(C)**, and F **(D)** substrates. **E)** Protease activity of 3CL^pro^ S2 pocket substitution mutants. “49” represents the substitution of 41-54 loop. “189” represents the substitution of 187-190 loop. αHA, antihemagglutinin; IB, immunoblotting. Protease activity was only restored in the double-loop substitution mutants of PEDV and PDCoV, therefore, substrate selectivity was subsequently determined exclusively for these two 3CL^pro^s. **F-G)** Diagram of the intra-molecular interactions in the S2 pocket of PDCoV **(F)** and PEDV **(G)** double-loop substitution mutants. **H)** The hydrogen bond occupancy of the intra-molecular interactions in the S2 pocket of PEDV and PDCoV double-loop substitution mutants.

**Figure S4**


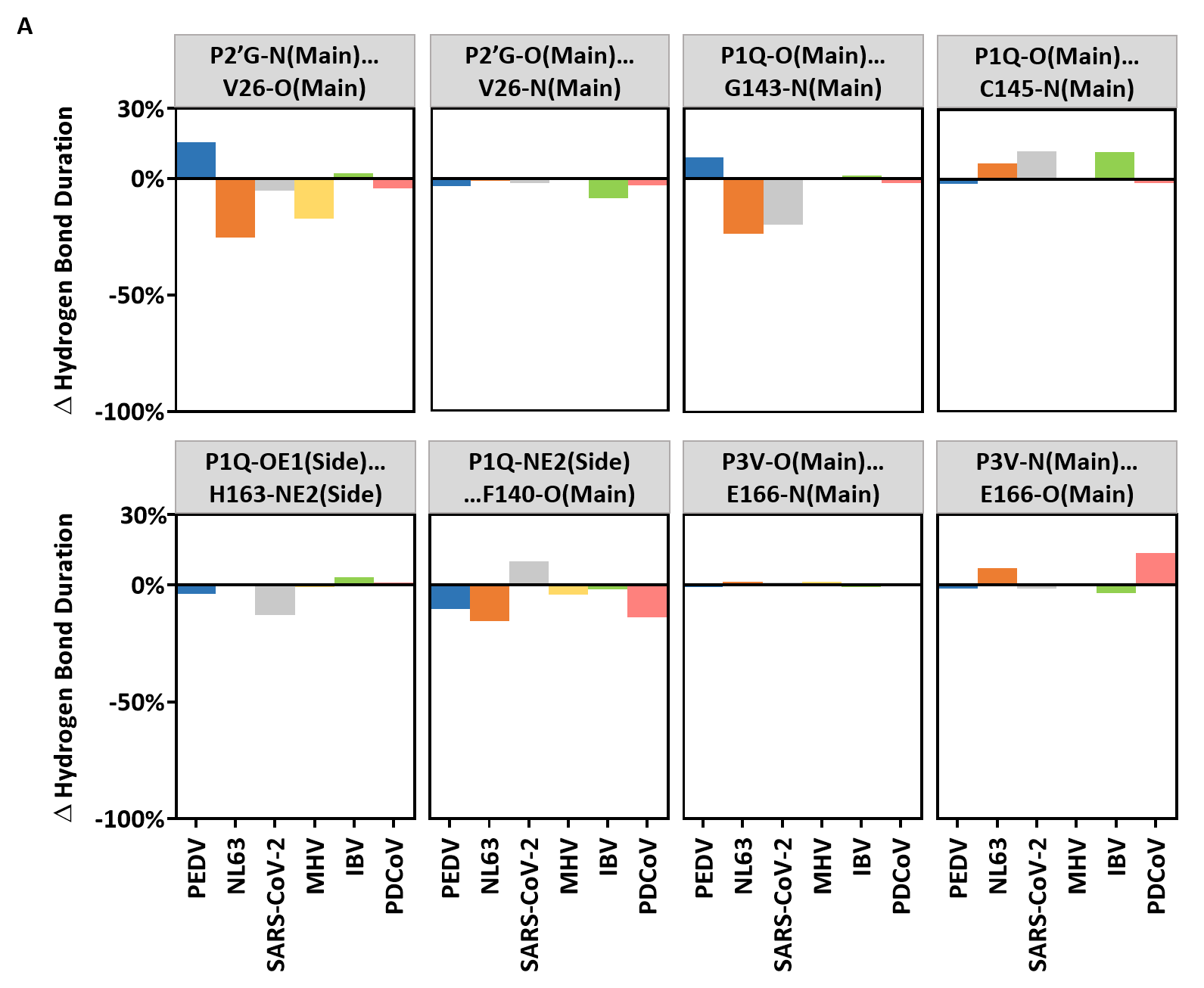


**Figure S4.** No significant variation of the hydrogen bond occupancy in P3, P1 and P2′ sites for P4-P substitution system relative to the WT system.

**Figure S5**

**
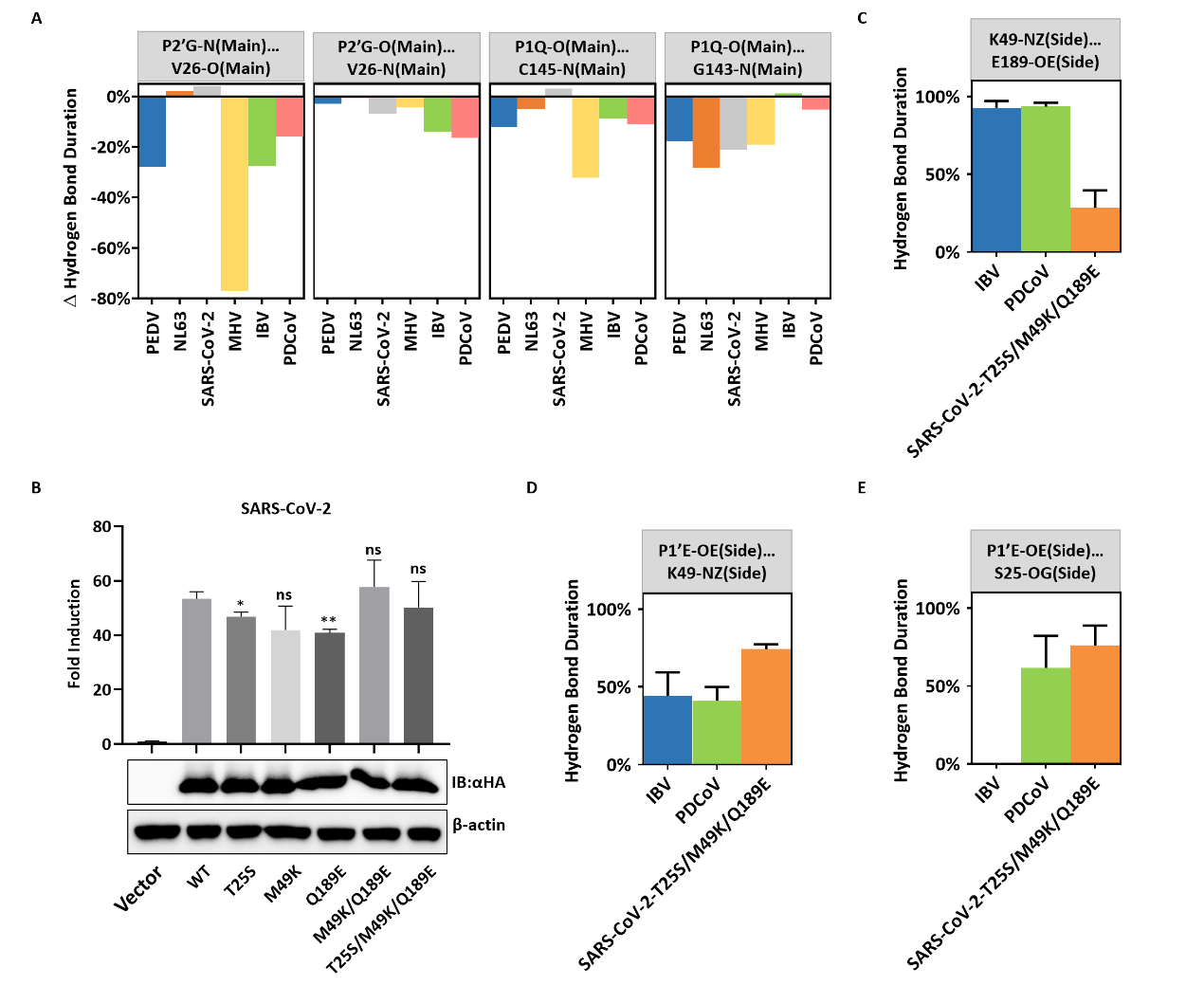
Figure S5.** Residue 49 in the S2 pocket govern the capacity of 3CL^pro^ to recognize and cleave P1′-E substrate. **A)** Variation of the hydrogen bond occupancy at P1 and P2′ sites in P1′-E substitution system relative to P1′-S WT system. **B)** Protease activity of a series of SARS-CoV-2 3CL^pro^ mutants. αHA, antihemagglutinin; IB, immunoblotting. **C)** The hydrogen bond occupancy between K49 and E189 in IBV, PDCoV, and modified SARS-CoV-2 3CL^pro^ systems. **D)** The hydrogen bond occupancy between K49 and P1′-E in IBV, PDCoV, and modified SARS-CoV-2 3CL^pro^ systems. **E)** The hydrogen bond occupancy between S25 and P1′-E in IBV, PDCoV, and modified SARS-CoV-2 3CL^pro^ systems.

**Figure S6**


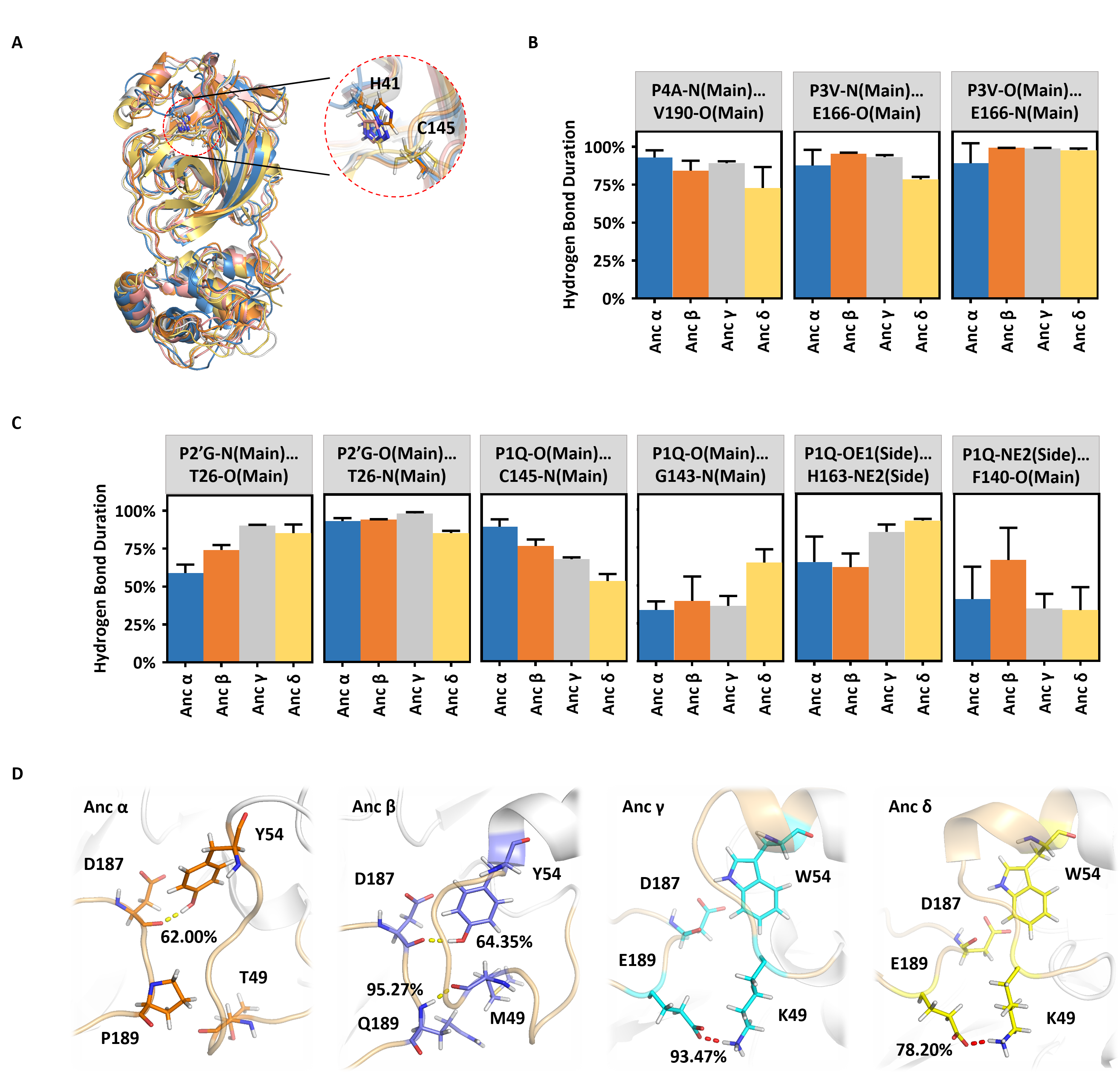


**Figure S6.** The four ancestral 3CL^pro^s possess the conserved and specific substrate-binding mode. **A)** Schematic overview of superimposed domains of one monomer from the four ancestral 3CL^pro^s to SARS-CoV-2 3CL^pro^. Red dotted circles indicate conserved Cys-His catalytic dyad. **B-C)** The hydrogen bond occupancy between the four ancestral CoV 3CL^pro^s and P3, P4 **(B)**, P1, or P2′ **(C)** site. **D)** Diagram of the three different intra-molecular interactions in the four ancestral CoV 3CL^pro^ S2 pockets. Percentage represents the hydrogen bond occupancy of the intra-molecular interactions in the S2 pocket.

**Figure S7**


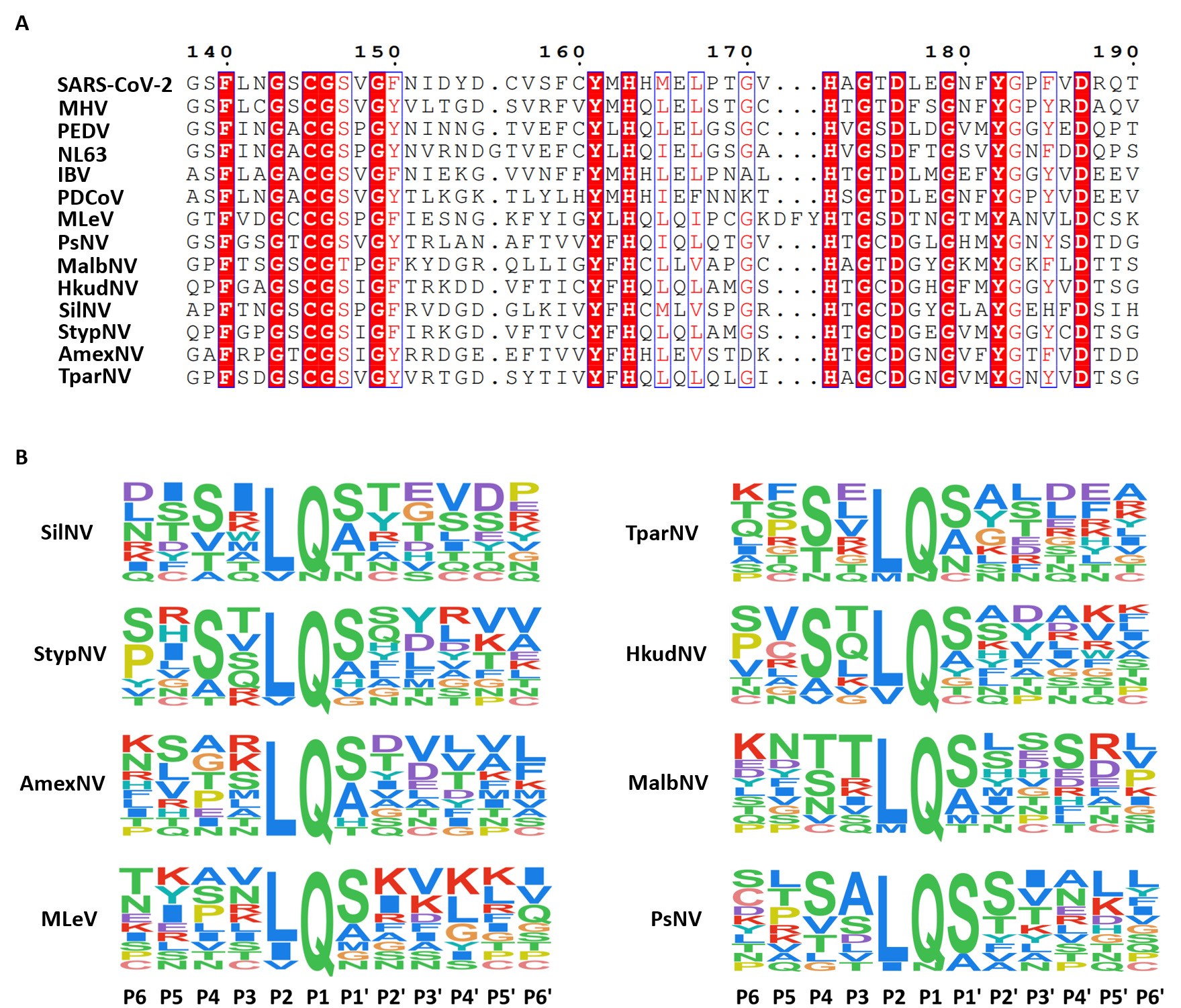


**Figure S7.** The newly discovered 3CL^pro^s exhibit a substrate-binding mode similar to that of modern 3CL^pro^s. **A)** The sequence alignment of the amino acid of the eight newly identified 3CL^pro^s and the six selected 3CL^pro^s. **B)** Amino acid sequence logos of the auto-cleavage sequences were created using PSSMSearch, and the height of the letters represents the relative frequency of the amino acid.

**Figure S8**

**
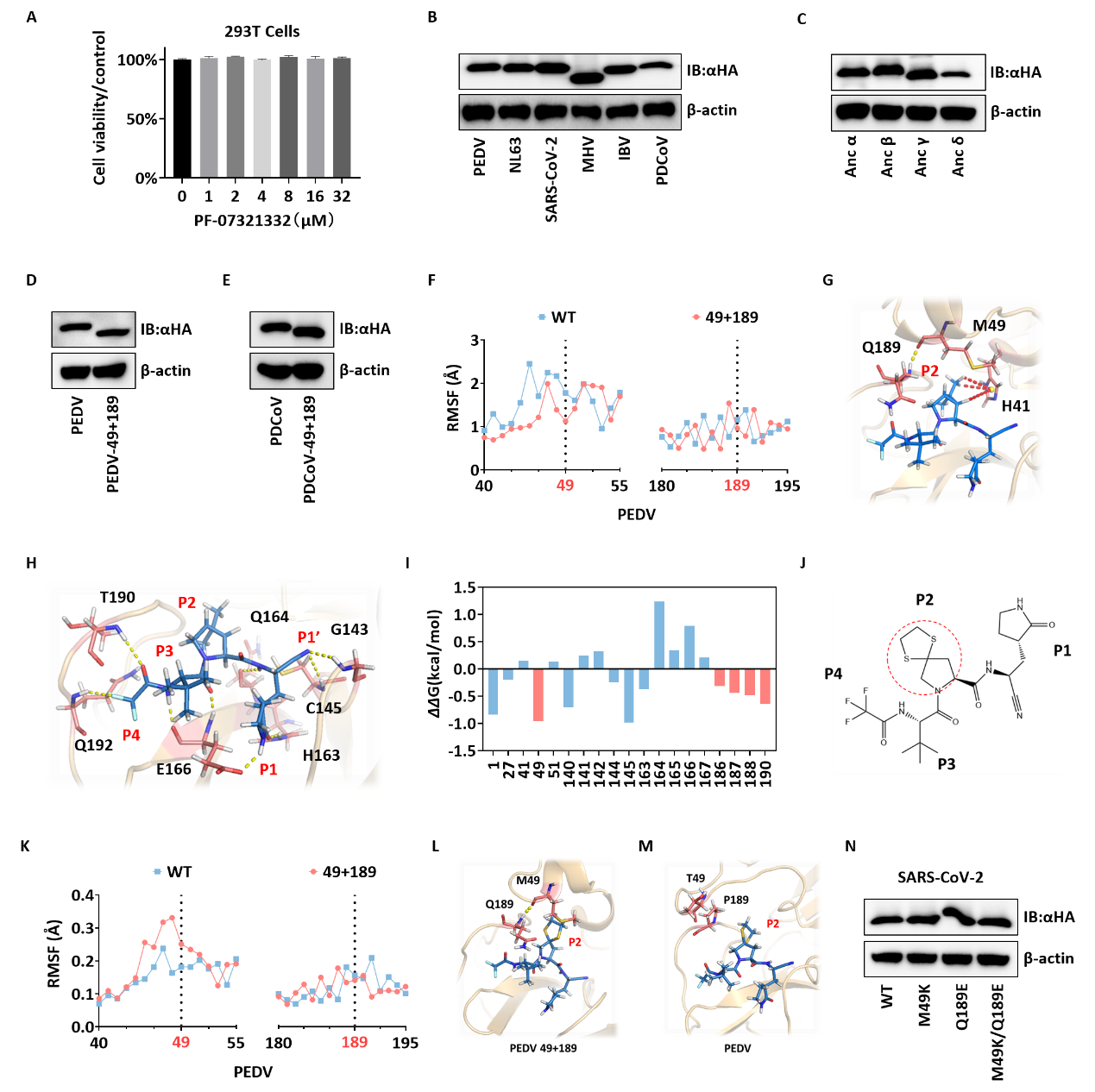
**

**Figure S8.** The specific S2 pocket is responsible for the more pronounced inhibitory effect of PF-07321332 towards β-CoV 3CL^pro^. **A)** 293T cells were treated with different concentrations of PF-07321332 for 12 hours, and then evaluated for cell viability by CCK-8 assay. The proliferation of 293T cells showed no substantial alteration in 32 μM. **B-E)** Detection of the expression of each 3CL^pro^ in inhibition experiment. **(B)** for the six selected 3CL^pro^s; **(C)** for the four ancestral 3CL^pro^s; **(D)** for PEDV 3CL^pro^ WT and mutant; **(E)** for PDCoV 3CL^pro^ WT and mutant. αHA, antihemagglutinin; IB, immunoblotting. **F)** RMSF of 40-55 loop and 180-195 loop in PEDV 3CL^pro^ WT and double-loop substitution mutant when binding PF-07321332. **G-H)** Diagram of the inter-molecular interactions between PEDV 3CL^pro^ 49+189 double-loop substitution mutant and PF-07321332. Blue color indicates PF-07321332. P4, P3, P1 motifs mainly contributed to electrostatic interactions in the active site to obtain selectivity **(H)**. The 6, 6-dimethyl-3-azabicyclo [3.1.0] hexane at P2 site effectively filled the lipophilic S2 pocket formed by M49, L169, H41, and Q189 **(G)**. **I)** Variation of binding affinity between PEDV 3CL^pro^ double-loop substitution mutant and PF-07321332 compared to PEDV WT 3CL^pro^. The red bar represents the amino acid in the S2 pocket that contributes to the increased binding free energy. **J)** Chemical structures of Simnotrelvir. **K)** RMSF of 40-55 loop and 180-195 loop in PEDV 3CL^pro^ WT and double-loop substitution mutant when binding Simnotrelvir. **L)** Diagram of the S2 pocket in PEDV 3CL^pro^ 49+189 mutant system. **M)** Diagram of the S2 pocket in PEDV 3CL^pro^ WT system. **N)** Detection of the expression of SARS-CoV-2 3CL^pro^ mutants in inhibition experiment. αHA, antihemagglutinin; IB, immunoblotting.

**Figure S9**

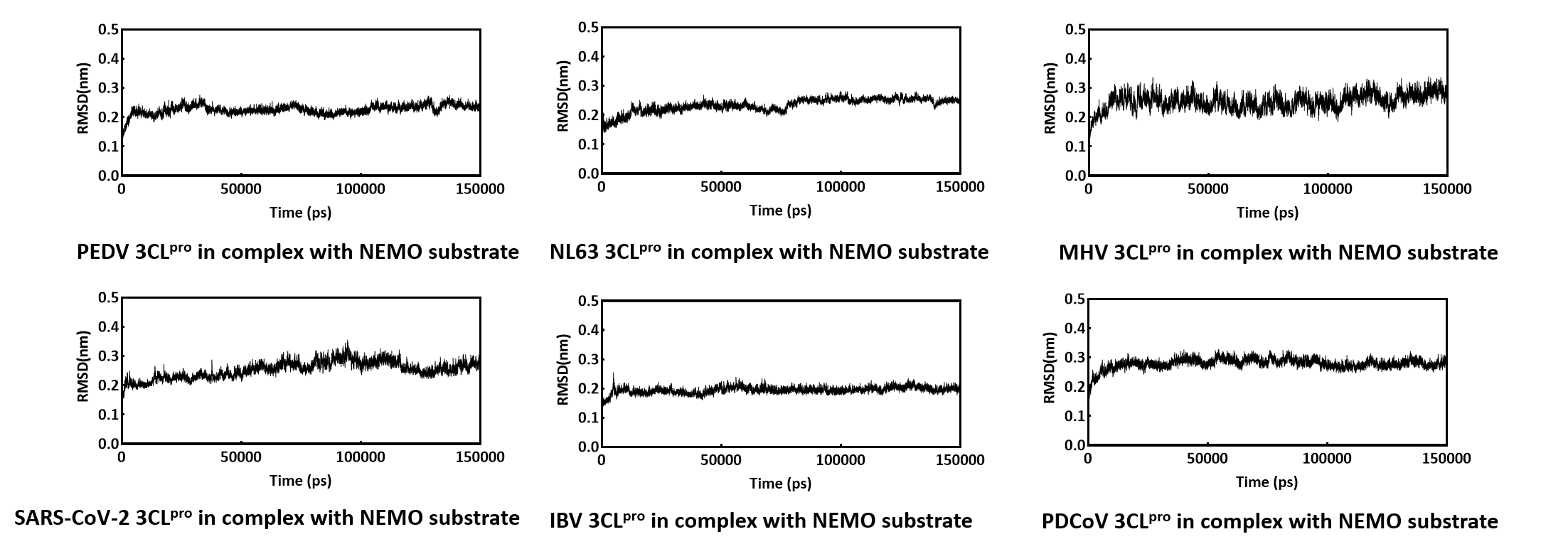

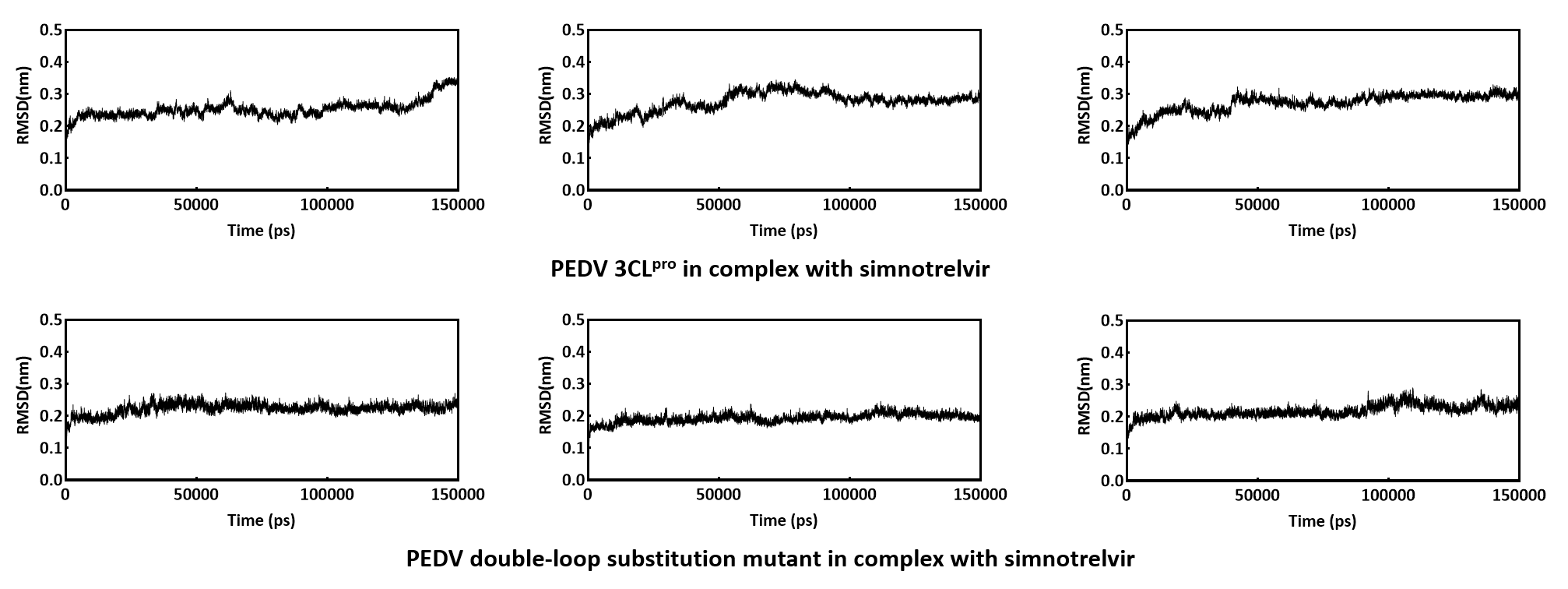


**Figure S9.** Plot of the RMSD values obtained for the systems. The RMSD has been computed using all non-hydrogen atoms taking as reference the structure prepared for the equilibrium simulations.

**Figure S10**

**A**

**B**

**Figure S10.** Distance distributions between residue 49 (A) or 189 (B) and P2-Leu in SARS-CoV-2 nsp4/5 substrate systems. Red, blue and black colors represent three repetitions. The time-dependent changes in the distances between amino acids 49/189 and P2-Leu in the WT system are consistent with the stability of hydrogen bonds in S2 pocket.
